# Integrating co-expression network analysis and machine learning to reveal the regulatory landscape of *GPD* genes in *Chlamydomonas reinhardtii* under salinity stress

**DOI:** 10.7717/peerj.21060

**Published:** 2026-04-14

**Authors:** Jorge A. Tzec-Interian, Santy Peraza-Echeverria, Virginia Aurora Herrera-Valencia, Elsa B. Gongora-Castillo

**Affiliations:** 1 Unidad de Biotecnología, Centro de Investigación Científica de Yucatán, Merida, Yucatan, Mexico; 2 Secihti-Departamento de Recursos del Mar, Centro de Investigación y de Estudios Avanzados del Instituto Politécnico Nacional, Merida, Yucatan, Mexico

**Keywords:** *Chlamydomonas reinhardtii*, Salinity stress, Transcriptional regulation, GPD genes, RNA-seq, WGCNA, Co-expression networks, Machine learning

## Abstract

**Background:**

Salinity stress imposes major constraints on cellular homeostasis. In *Chlamydomonas reinhardtii,* glycerol biosynthesis is a key osmoprotective response mediated by glycerol-3-phosphate dehydrogenases (GPDs). Although *GPD2* and *GPD3* genes are known to be salt-responsive with high sequence identity and shared metabolic roles, the regulatory mechanisms of their differential expression have remained unclear. Here, we leverage publicly available time-course RNA-seq data to dissect the transcriptional regulation of these genes, using an integrative network analysis and machine-learning framework.

**Methods:**

Weighted Gene Co-expression Network Analysis (WGCNA) was applied to transcriptomes of *C. reinhardtii* exposed to salinity stress. Modules containing *GPD2* and *GPD3* genes were analyzed for functional enrichment, transcription factor associations, and *cis*-regulatory elements. Module robustness was independently evaluated using a Random Forest classifier to assess the separability of gene-to-module assignments.

**Results:**

*GPD2* and *GPD3* genes clustered into distinct co-expression modules with contrasting temporal profiles. *GPD2* was associated with early stress responses and co-expressed with *bZIP* and *C2C2-GATA* transcription factors, whereas *GPD3* was linked to later adaptive responses and associated with *MYB*, *SBP*, and *ALFIN*-like transcription factors. These differences were supported by distinct *cis*-regulatory motifs. Random Forest validation confirmed strong module coherence, providing independent support for the inferred regulatory programs.

**Conclusions:**

Despite their high sequence similarity, *GPD2* and *GPD3* are embedded in temporally and regulatory distinct transcriptional networks during salinity stress. By integrating co-expression analysis with supervised machine learning for internal module validation, this study introduces a robust strategy to refine regulatory inference from transcriptomic data. More broadly, it highlights the value of data reuse and integrative analysis for uncovering regulatory divergence among genes, with potential implications for functional optimization in microalgae and related biological systems.

## Introduction

The unicellular green alga *Chlamydomonas reinhardtii* is a well-established model for studying transcriptional responses to abiotic stress facilitated by its comprehensive genomic resources (Merchant et al., 2007; Salomé & Merchant, 2019; Craig et al., 2023).

Salinity is a significant stress explored in this model as NaCl exposure induces notable physiological and transcriptional changes, including growth inhibition, reactive oxygen species accumulation, and increased synthesis of glycerol (Shetty, Gitau & Maróti, 2019; Bazzani et al., 2021).

Glycerol biosynthesis is a key osmoprotective response to salinity stress and involves glycerol-3-phosphate dehydrogenases (GPDs) enzymes, which generate glycerol-3-phosphate as a precursor for both glycerol and triacylglycerol (TAG) (Casais-Molina et al., 2016; Oren, 2017). The *C. reinhardtii* genome encodes five *GPD* genes (*GPD1–GPD5*), of which *GPD2* and *GPD3* are consistently up-regulated under saline conditions (Casais-Molina et al., 2016; Morales-Sánchez et al., 2017; Zhang et al., 2022). Although these genes share high sequence similarity and participate in the same metabolic pathway, their mechanisms underlying their transcriptional regulation during salt stress remain poorly understood. A deeper understanding of how *GPD* are differentially regulated may therefore support the development of biotechnological strategies to optimize osmoprotection and lipid-related pathways in *C. reinhardtii* and other biologically relevant systems.

Weighted Gene Co-expression Network Analysis (WGCNA) is an effective strategy to identify modules of co-expressed genes and inferring functional associations (Zhang & Horvath, 2005). In *C. reinhardtii*, co-expression network analyses have been used to map global transcriptomic responses to various stresses, revealing key regulators and coordinated modules (Gargouri et al., 2015; López García De Lomana et al., 2015; Romero-Campero et al., 2016; Salomé & Merchant, 2021; Zhang et al., 2022). However, the high dimensionality of transcriptomic datasets often makes it difficult to interpret the internal structure of the WGCNA modules or determine genes that strongly contribute to the module organization hiding the biologically relevant subgroups. To address this limitation, supervised machine learning approaches provide a validation layer to assess the separability and coherence of WGCNA-derived modules. In particular, Random Forest classifiers offer a robust means to evaluate module robustness by testing whether genes assigned to the same module can be reliably classified based on their expression patterns. The integration of co-expression network analysis with supervised machine learning has been increasingly applied as an effective strategy to strengthen the interpretability and confidence of network-based inferences in transcriptomic studies highlighting key regulatory candidates within complex biological systems (Conn et al., 2019; Auslander, Gussow & Koonin, 2021; Zhu, Yang & Zu, 2022; Kong et al., 2023).

In this study, we investigate the transcriptional regulation of the genes *GPD2* and *GPD3* in *C. reinhardtii* under salinity stress using an integrative framework that combines co-expression network analyses with supervised machine learning. Our results revealed that despite their shared metabolic role, *GPD2* and *GPD3*, are regulated under distinct context defined by specific transcription factors and associated *cis*-regulatory elements. To support these findings, we apply a Random Forest-based approach to assess the robustness of gene-to-module assignments, providing an internal validation framework tailored to the analysis of a specific gene family. Together, these results offer new insights into the regulatory mechanisms underlying glycerol biosynthesis during salt stress.

## Materials & Methods

### Data acquisition and RNA-seq analysis

Publicly available RNA-seq data from a time-course experiment involving *C. reinhardtii* under 200 mM NaCl treatment (Zhang et al., 2022) was obtained from the Sequence Read Archive (SRA) (bioproject ID: PRJNA770825). A total of 24 RNA-seq libraries comprising 8 time points (0, 2, 4, 8, 12, 24, 48, and 72 h), each with three replicates, were downloaded and quality-checked using FastQC (v0.11.9) (Andrews, 2010). Although the original dataset included a 96 h time point, this stage was excluded from our analysis because salinity-induced transcriptional changes in *C. reinhardtii* occur predominantly before 72 h (Zhang et al., 2022). Low-quality sequences and primers were filtered and trimmed using Trimmomatic (v0.38) (Bolger, Lohse & Usadel, 2014). Pre-processed sequences were aligned to the *C. reinhardtii* reference genome v5.6 (Merchant et al., 2007) using HISAT2 (v2.2.0) with default settings (Kim, Langmead & Salzberg, 2015). The reference genome was obtained from Phytozome v13 (Goodstein et al., 2012). SAM files were converted and sorted to BAM format using SAMTools (v1.8) (Li et al., 2009), and expression matrices were generated using FeatureCounts from subread (v2.0.1) assigning uniquely mapped reads (Liao, Smyth & Shi, 2014).

### Filtering, normalization and outlier detection

The expression matrix was filtered by removing genes with ≤10 average reads across all samples. Variance stabilization transformation (VST) was applied using DESeq2 (v1.38.3) (Love, Huber & Anders, 2014). Principal component analysis (PCA) was performed to assess replicate structure, and outlier detection was carried out on VST-normalized data using hierarchical clustering of pairwise Pearson’s correlation, following standard WGCNA guidelines (Langfelder & Horvath, 2008; Horvath, 2011). Individual gene expression profiles were plotted using log-transformed Transcripts per Million (logTPM).

### Differential expression analysis

Differential expression analysis was performed on 22 filtered samples (3 controls and 19 NaCl-treated) using DESeq2 (v1.38.3) (Love, Huber & Anders, 2014). Differentially expressed genes (DEGs) were identified using Log_2_FC ≥ 2 and *α* = 0.05 with Benjamini–Hochberg *p*-value adjustment. Two comparisons were conducted: control *vs.* all NaCl-treated samples across time points, and control *vs.* NaCl-treated samples at each specific time point (2, 4, 8, 12, 24, 48, and 72 h). Gene identifiers from *C. reinhardtii* genome v5.6 were used for annotation (Merchant et al., 2007). Volcano plots were generated using EnhancedVolcano (v1.24.0) (Blighe, Rana & Lewis, 2024), and common/unique up- and down-regulated genes were visualized using UpSetR (v1.4.0) (Conway & Gehlenborg, 2019).

### Construction of weighted gene co-expression network

The co-expression network was constructed using WGCNA (v3.3.2) (Langfelder & Horvath, 2008) on the complete filtered and normalized gene expression dataset (22 samples, 15,323 genes). The soft threshold (*β* value) was determined by assessing scale independence and mean connectivity over power values 1–30. Power value 16 was selected (R^2^ > 0.8) with mean connectivity of 42. Modules were constructed using blockwiseModules with minimum module size of 30, merge cut height of 0.25, TOMType “signed”, and deepSplit of 4. The network type “signed hybrid” was selected as WGCNA documentation suggests to focus on positive co-expression relationships (Zhang & Horvath, 2005; Langfelder & Horvath, 2008). The grey module (unassigned genes) was excluded from downstream analysis. Hub genes were identified using module membership (kME) and connectivity (kIM) values calculated with intramodularConnectivity.fromExpr (Langfelder, Mischel & Horvath, 2013), and annotated using *C. reinhardtii* v5.6 (Merchant et al., 2007).

### Enrichment analysis for gene ontology and metabolic pathway analysis

Gene ontology enrichment analysis was conducted using TopGO package (v2.62.0) (Alexa & Rahnenfuhrer, 2025) to identify biological processes and molecular functions in gene modules. GO terms were obtained from Phytozome using BiomaRt (v2.54.1) (Durinck et al., 2009). Analysis was performed using the “Weigh01 algorithm” and Fisher’s test with Benjamini–Hochberg *p*-value adjustment. For modules containing *GPD* genes, the top five significant biological processes and functions were identified.

Additionally, genes related to glycerol and TAG pathways into *GPD2* and *GPD3* modules were mapped using Phytozome v13 database (Goodstein et al., 2012), targeting genes annotated in lipid metabolism pathways containing keywords “lipid,” “glycerol,” and “lipases.” The biomaRt package (v2.60.1) was used to connect to Ensembl Plants database and access *C. reinhardtii* dataset (Merchant et al., 2007; Durinck et al., 2009). Genes and associated pathways were retrieved using getBM, extracting Ensembl gene ID, external gene name, and Kyoto Encyclopedio of Genes and Genomes (KEGG) enzyme ID (Kanehisa & Goto, 2000; Kanehisa et al., 2023).

### *GPD* gene-centered network and regulatory analysis

Modules containing *GPD2* and *GPD3* were selected for detailed analysis of co-expression relationships, protein domains, transcription factor interactions, and promoter regions. The topological overlap matrix (TOM) was used to extract edges and nodes using exportNetworkToCytoscape from WGCNA (v3.3.2) (Langfelder & Horvath, 2008), with a threshold of 0.02 for weighted interactions. Co-expression networks of magenta and black modules were reconstructed in Python (v3.12.4) using NetworkX (v3.4.2) (Hagberg et al., 2008), filtering edges by weight ≥ 0.17.

Protein domain annotation was performed over uncharacterized genes directly co-expressed with *GPD2* and *GPD3* genes using InterProScan (v5.59–91.0) (Jones et al., 2014) on amino acid sequences retrieved from *C. reinhardtii* genome v5.6 (Phytozome v13) (Merchant et al., 2007; Goodstein et al., 2012). GPD protein sequences were corroborated with genome v6.1 (Craig et al., 2023) and aligned using Clustal Omega (v1.2.4) *via* EMBL-EBI platform (Sievers & Higgins, 2014), with conserved domains mapped onto the alignment.

For transcription factor analysis, a curated list of *C. reinhardtii* transcription factors was obtained from PlantTFDB (v5.0) (Tian et al., 2020) and cross-referenced with genes in *GPD*-containing modules. Promoter regions (two kb upstream of transcription start site) of *GPD2* and *GPD3* were analyzed for *cis*-regulatory elements (CREs) associated with abiotic stress responses using PlantCARE database (Lescot et al., 2002). Promoter sequence conservation was assessed by multiple sequence alignment using Clustal Omega, with mismatches and gaps quantified in 25-bp sliding windows.

### Validation of differential expression analysis

A comparative analysis was performed using the results of an independent RNA-seq study of *C. reinhardtii* exposed to 100 mM NaCl (Vilarrasa-Blasi et al., 2022). Expression values were obtained from Table S1 of Vilarrasa-Blasi et al. (2022), which contains transcript-level quantification for 19,527 entries. From this dataset, transcripts with baseMean ≤ 5 or maximum —Log2FC—≤ 0.01 were excluded, retaining 12,159 transcripts. Transcript annotations were mapped to gene-level identifiers, and genes differentially expressed (Log2FC >2; adjusted *p*-value <0.05) at 4 h or 24 h in both datasets were selected, and their expression profiles across all time points were extracted to evaluate consistency under different salinity conditions.

Additionally, to assess RNA-seq data processing preservation of expected gene expression trends, previously published qPCR-based relative expression values were retrieved from Zhang et al. (2022). Eight experimentally validated genes were identified in *C. reinhardtii* genome by mapping the primers reported by Zhang et al. (2022) and RNA-seq-derived Log2FC values were compared with published qPCR results at selected time points (2 h, 12 h, 24 h, and 48 h). Pearson’s correlation analyses were conducted to evaluate consistency between datasets.

### Integration of qPCR-validated genes into co-expression modules

To further evaluate the biological relevance of the inferred network, four qPCR-validated genes that were differentially expressed under 200 mM NaCl from Zhang et al. (2022) study, were identified and mapped to their respective co-expression modules. The topological overlap matrix (TOM) was used to extract edges and nodes with the function exportNetworkToCytoscape from WGCNA (v3.3.2) (Langfelder & Horvath, 2008). Node degree (connectivity) was quantified at multiple weight thresholds (0.025, 0.05, 0.10, 0.15, 0.17, 0.20) to assess the robustness of gene connections within the network. Subnetworks were visualized with a minimum degree of 2, highlighting the placement and connectivity of qPCR-validated genes within their modules.

### Co-expression network validation using machine learning

The normalized expression dataset (15,323 genes × 22 samples) was used as input for Random Forest analysis (randomForest v4.7-1.2) (Breiman, 2001). For model development, the dataset was randomly split into training (80%) and test (20%) sets. Class imbalance across modules was addressed using upsampling (caret v7.0-1) (Kuhn, 2024). The model was trained using 500 trees and evaluated by 5-fold cross-validation. Performance metrics (accuracy, Cohen’s Kappa, and ROC curves) were computed with caret and pROC v1.18.5 (Robin et al., 2011), and UMAP projections (UMAP v0.2.10.0) (McInnes, Healy & Melville, 2018) were used to visualize module separability based on model predictions.

## Results

### Salinity effect on *GPD* genes expression

*Chlamydomonas reinhardtii* genome contains five *GPD* genes. These genes are characterized by containing a conserved NAD-dependent glycerol-3-phosphate dehydrogenase domain. Additionally, *GPD2, GPD3*, and *GPD4* possess haloacid dehalogenase-like hydrolase (HAD-like) and phosphoserine phosphatase domains. *GPD2* and *GPD3* are genes with high sequence similarity and related metabolic functions (Fig. S1 in File S1) (Herrera-Valencia et al., 2012; Casais-Molina et al., 2016; Morales-Sánchez et al., 2017).

We analyzed publicly available RNA-seq data from *C. reinhardtii* under salt stress to perform differential expression analysis and confirm the upregulation of *GPD2* and *GPD3* (Zhang et al., 2022). High-quality reads from 24 samples were mapped to the *C. reinhardtii* reference genome (version 5.6) (Merchant et al., 2007), yielding 90.28% ± 0.29% alignment rate and 83.73% ±  2.23% read assignment rate, resulting in a matrix with 17,741 genes and 24 samples (Table S1). After filtering low-expression genes (2,418) (Fig. S1; File S2), PCA of variance-stabilized expression values showed overall consistency among biological replicates (Fig. S2A). In contrast, hierarchical clustering identified two outlier samples that were removed (Fig. S2C). The resulting dataset comprised 22 samples and 15,323 genes and revealed a clear time-dependent transcriptional structure, separating early (2–8 h) and prolonged (12–72 h) responses into distinct clusters (Fig. S2E).

Differential expression analysis revealed 252 up-regulated and 136 down-regulated genes under NaCl treatment (Fig. S3; File S3). Across the time course, up-regulation peaked at 8 h (945 genes, 6.2%) and 2 h (830 genes, 5.4%), whereas down-regulation was most pronounced at 2 h and 4 h (725 genes, 4.7% each) and remained high at 8 h (714 genes, 4.7%), identifying 8 h as a key transcriptional inflection point in the salinity response (Fig. S4).

**Figure 1:**
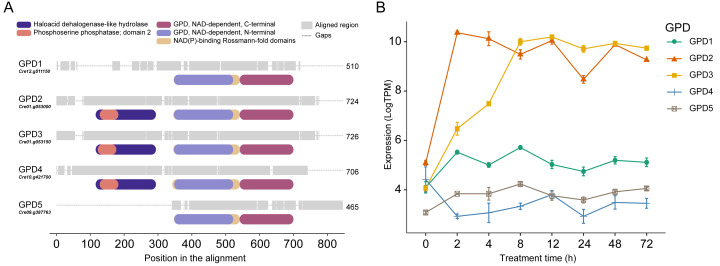
Glycerol-3-phosphate dehydrogenase (*GPD*) genes in *C. reinhardtii*. (A) Multiple alignment of five nuclear-encoded *GPD* isoforms showing amino acid length (numbers), aligned regions (gray bars), and conserved domains (colored bars). (B) *GPD* expression under 200 mM NaCl from 0–72 h. Expression levels in LogTPM show mean values ±  standard deviation.

When analyzing the expression of five *GPD* genes under salinity treatment, only *GPD2* and *GPD3* were significantly induced (Log_2_FC = 4.21 and 5.06, respectively), whereas the remaining *GPD* genes were unchanged (Fig. 1B, Fig. S3). *GPD2* peaked early at 2 h (Log_2_FC = 4.8), while *GPD3* exhibited a delayed, sustained induction peaking at 12 h (Log_2_FC = 5.79) (Fig. S5), showing that *GPD2* and *GPD3* respond to NaCl through distinct temporal transcriptional programs.

To assess the reproducibility of the transcriptional response capture by our analysis, we implemented two complementary validation strategies based on independent datasets. First, we compared our differential expression results with those reported in an independent RNA-seq study of *C. reinhardtii* exposed to similar salinity stress (100 mM NaCl) (Vilarrasa-Blasi et al., 2022). This comparison identified 40 consistently up-regulated genes and 63 down-regulated genes at shared time points (4 h and 24 h), with highly concordant expression profiles between studies (Fig. S6), supporting the reproducibility of the salt-responsive transcriptional program. Second, to assess the integrity of our data processing pipeline, we compared our RNA-seq expression estimates with published quantitative PCR (qPCR) measurements for 8 salt-responsive genes validated experimentally by Zhang et al. (2022). This analysis showed strong correlations at early stages of the response (2 h: *r* = 0.93, *p* = 0.00068; 12 h: *r* = 0.91, *p* = 0.0016), and moderate but consistent correlations at later time points (24 h: *r* = 0.61; 48 h: *r* = 0.56) (Fig. S7). Together this evidence indicates that our analytical framework robustly captures the temporal dynamics of gene expression during the salinity stress and is consistent across independent datasets.

### Genes associated with *GPD* genes and functional identification

**Figure 2:**
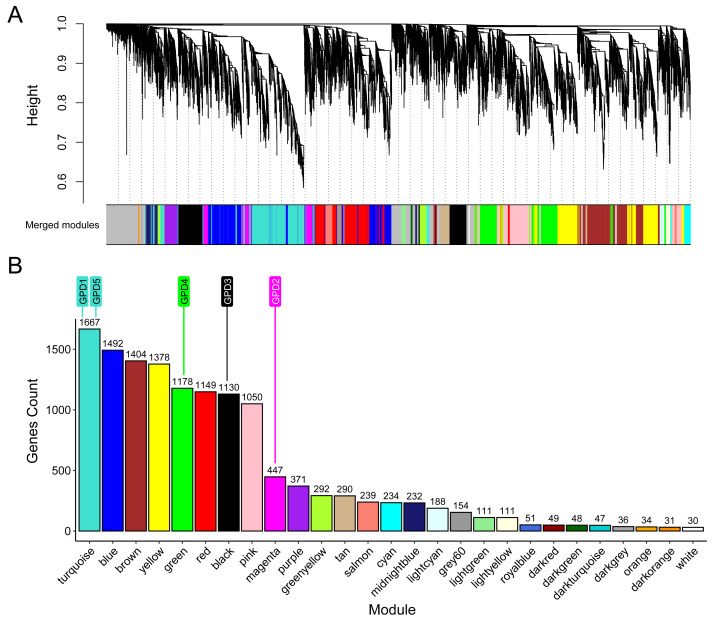
Weighted gene co-expression network analysis (WGCNA) of *C. reinhardtii* transcriptome under 200 mM NaCl treatment. (A) Dendrogram of gene clustering with merged modules based on module similarity. (B) Distribution of number of genes per module and *GPD* gene’s location.

To identify genes associated with the *GPD* genes during the salinity response in *C. reinhardtii*, a WGCNA was performed with 15,323 genes across 22 samples (Fig. S8), resulting in 28 modules (Fig. 2). The largest modules were turquoise (1,667 genes), blue (1,492 genes), and brown (1,404 genes), while the smallest were orange (34 genes), dark orange (31 genes), and white (30 genes) (Fig. 2B). *GPD* genes were distributed across different modules: *GPD1* and *GPD5* in turquoise, *GPD2* in magenta (447 genes), *GPD3* in black (1,130 genes), and *GPD4* in green (1,178 genes). Notably, 18 genes in the magenta module (including *GPD2*) and 19 genes in the black module (including *GPD3*) were up-regulated, with most differentially expressed genes in these modules being up-regulated (Table S2, Fig. S9).

**Figure 3:**
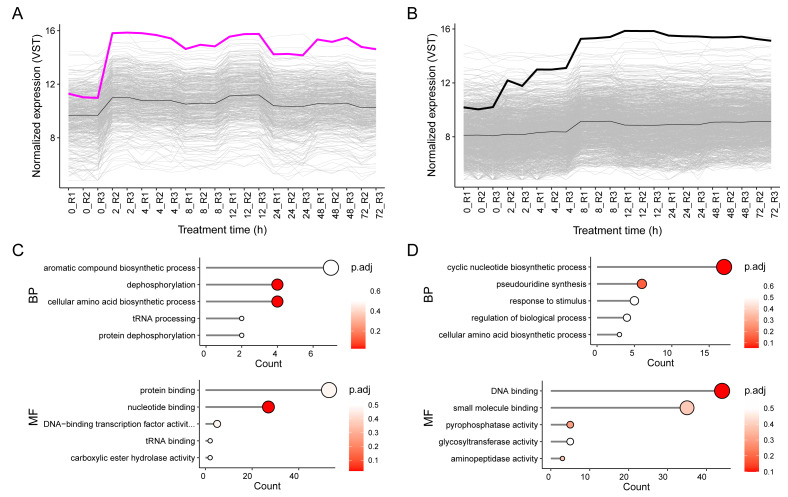
Expression profiles and Gene Ontology (GO) annotation of magenta and black modules under salt stress. (A) Expression profile of *GPD2* (bold line) in magenta module. (B) Expression profile of *GPD3* (bold line) in black module. Module means are represented in dashed lines, and other genes in gray lines. (C) Top five GO Biological Process (BP) and Molecular Functions (MF) enriched in magenta. (D) Top five GO Molecular Function (BP) and Molecular Functions (MF) enriched in black module.

GO enrichment analysis was performed revealing a total of 21 biological processes (BP) and 25 molecular functions (MF) (Table S3). Of these, the magenta module (*GPD2*) was enriched in dephosphorylation (four genes), and amino acid biosynthesis (four genes) BP, and tRNA binding, protein binding (53 genes), nucleotide binding (27 genes), and DNA-binding transcription factor activity (five genes) MF (Fig. 3), indicating *GPD2*’s involvement in a transcriptionally active regulatory network. While, the black module (*GPD3*) was enriched in cyclic nucleotide biosynthesis (17 genes), pseudouridine synthesis (seven genes), regulation (five genes), and response to stimulus (six genes) BP, and small molecule binding (35 genes), pyrophosphatase activity (five genes), glycosyltransferase activity (five genes), and DNA binding (44 genes) MF (Fig. 3), underscoring its role in transcriptional and metabolic responses to salinity.

In contrast, *GPD1* and *GPD5*, located in the turquoise module, were co-expressed with genes enriched in transcriptional regulation functions (DNA binding, nucleotide binding, RNA modification) (Table S3), suggesting participation in broad regulatory processes during stress, while *GPD4*, in the green module, exhibited moderate down-regulation (Log2FC = −1.59) (Fig. S5) and was co-expressed with potassium and phosphate transmembrane transport genes (Table S3). Notably, the green module contained 11–23% of down-regulated genes across time points (∼150–250 genes; Fig. S9), indicating selective repression of transport-associated functions during salinity response.

### *GPD2* and *GPD3* connections in the network during salinity stress

**Figure 4:**
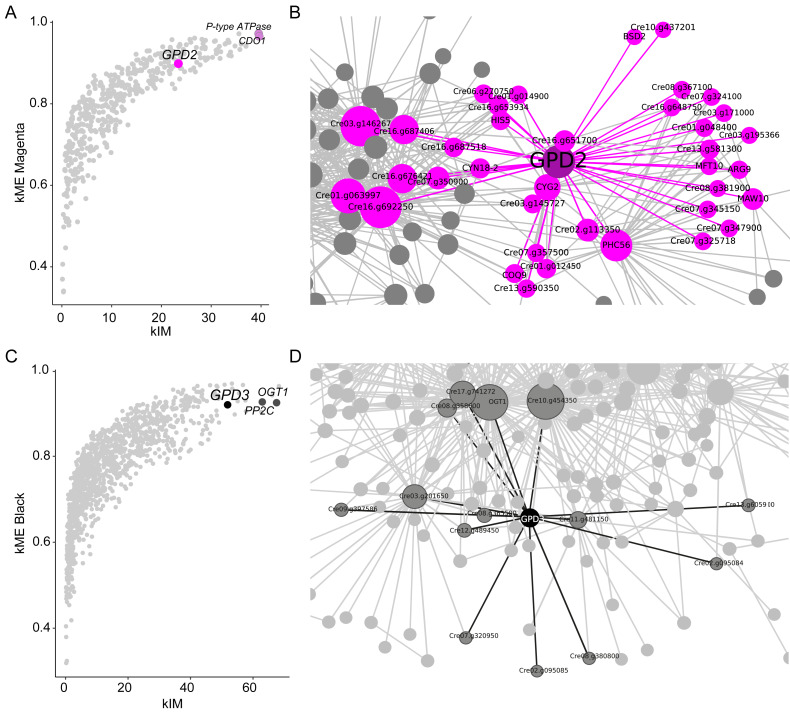
Connectivity of *GPD2.* and *GPD3* in the *C. reinhardtii* transcriptome under salt stress. (A) Module eigengene-based connectivity (kME) *versus* intramodular connectivity (kIM) for *GPD2* and hub genes (*CDO1*, P-type ATPase) in the magenta module. (B) Co-expression network of *GPD2* in magenta module. (C) kME *vs.* kIM for *GPD3* and hub genes (*OGT1*, *PP2C*) in the black module. (D) Co-expression networks of *GPD3* in the black module.

To explore the regulatory context of *GPD2* and *GPD3* within their respective modules, we identified hub genes characterized by high module membership (kME) and strong intramodular connectivity (kIM) (Langfelder & Horvath, 2008) (Fig. 4). In the magenta module, the top hub was a putative cation-transporting *P-type ATPase* (*Cre09.g410050*) (kME = 0.97, kIM = 39.516), followed by cysteine dioxygenase *CDO1* (*Cre03.g174400*) (kME = 0.96, kIM = 39.634). Both hub genes were differentially expressed compared to the control after 2 h of NaCl treatment (Log2FC >2; p.adj <0.05), with *CDO1* remaining up-regulated until 12 h, suggesting active participation in the early transcriptional response to salinity stress. *GPD2* was ranked 59th out of 447 genes in the magenta module (kME = 0.89; kIM = 23.401) (Fig. 4A, Fig. S3).

In the black module, O-linked N-acetylglucosamine transferase *OGT1* (*Cre12.g552851*) (kME = 0.92, kIM = 67.551) and phosphatase 2C-related protein *PP2C* (Cre10.g454450) (kME = 0.92, kIM = 62.977) were identified as hub genes. *OGT1* was up-regulated (Log2FC = 3.85, p.adj = 0.001), indicating active participation in the salinity response. *GPD3* ranked 14th out of 1,130 genes (kME = 0.91, kIM = 51.871) (Fig. 4C, Fig. S3), suggesting an important role in the structural and functional organization of the black module during salinity response.

Network analysis revealed *GPD2*’s direct connections to 37 genes out of 981 total connections in the magenta module (Fig. 4B, Fig. S10). Of these, 28 genes were functionally annotated and 10 were identified as up-regulated under salinity stress, including protein kinases (*Cre02.g113350, Cre03.g171000, Cre10.g43720, Cre07.g325718*), adenylate/guanylate cyclase *CYG2*, Major Facilitator Superfamily Transporter *MFT10*, and transcription factors (bZIP *Cre16.g692250, C2C2_GATA Cre03.g146267*, APRR1-related *Cre16.g676421*, Ankyrin repeats *Cre07.g350900*, zinc finger proteins *Cre01.g048400, Cre08.g381900*) (Table S4, Fig. S11), suggesting complex regulatory control of *GPD2* expression through kinase-mediated signaling and cyclic nucleotide pathways.

In contrast, *GPD3* showed direct connections to 14 genes out of 1,064 total connections in the black module (Fig. 4D, Fig. S12). Notably, we were able to identify by functional annotation only four genes including hub gene *OGT1* and CPG-binding protein/PHD-finger protein ALFIN-like 4 (*Cre07.g320950*) (Table S4). Protein domain functional annotation of uncharacterized genes revealed cytoskeleton-related domains in *Cre12.g489450* (calponin homology) and *Cre13.g605900* (WASP-interacting protein-related) (Fig. S13).

To further assess the biological relevance of the co-expression network, we mapped four salt-responsive genes that were experimentally validated by Zhang et al. (2022) and showed significant differential expression under salinity stress (Log_2_FC >1). This analysis allowed us to examine their modular assignment and connectivity within the network. Two of these genes, the transcription factors *Cre02.g103450* (MYB-related) and *Cre04.g228400* (WRKY), displayed consistently high node degrees within the blue module across multiple edge-weight thresholds (Table S5), indicating stable topological centrality. Although all four genes showed increased expression under salinity (Fig. S14), only these transcription factors were embedded within highly connected network neighborhoods. This observation underscores that the *GPD*-associated network captures biologically meaningful regulatory structure, in which highly connected nodes correspond to experimentally supported transcriptional regulators, thereby reinforcing that the inferred *GPD2* and *GPD3* connections are not only statistically supported but also functionally informative.

### Transcription factors associated with *GPD*2 and *GPD*3 genes and their *cis*-regulatory elements

**Figure 5:**
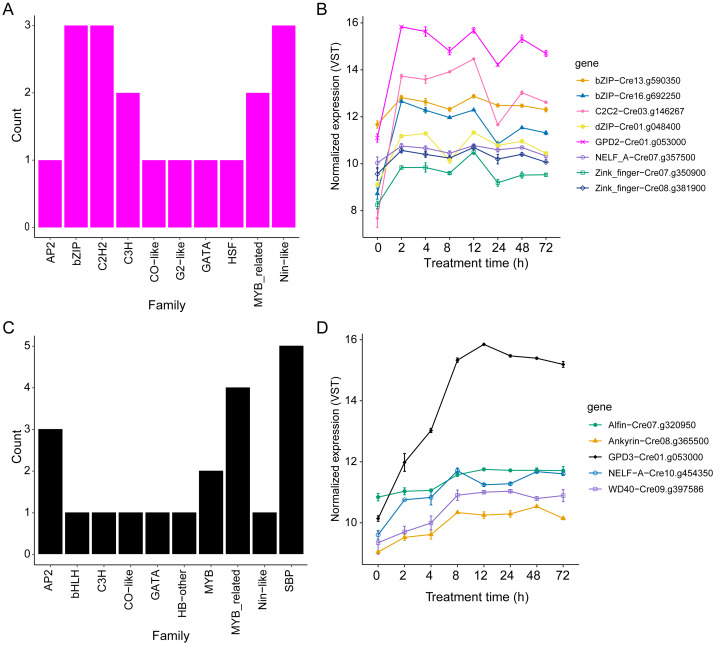
Distribution and expression profiles of transcription factors (TFs) co-expressed with *GPD2* and *GPD3* in the *C. reinhardtii* transcriptome under 200 mM NaCl treatment. (A) Frequency of TF families identified within the magenta module containing *GPD2*. (B) Expression patterns of *GPD2* and its connected TFs in the magenta module. (C) Frequency of TF families identified within the black module containing *GPD3*. (D) Expression patterns of *GPD3* and its connected TFs in the black module. Expression values correspond to variance-stabilizing transformation (VST)-normalized counts plotted across the time course.

To investigate potential differences in the regulation of *GPD2* and *GPD3* expression, transcription factors (TFs) were identified across modules (Figs. S15–S16). In the magenta module (*GPD2*), the predominant TF families were *bZIP, C2H2,* and *Nin-like*, each represented by three genes (Fig. 5A). Among the genes connected to *GPD2*, two *bZIP* TFs were identified, with *Cre16.g692250* standing out for its high connectivity (node degree = 66) (Table S4, Fig. 5B). Additional TFs linked to *GPD2* included an up-regulated *C2C2_GATA* TF with strong connectivity (node degree = 61), as well as three genes encoding DZIP-like finger proteins (*Cre07.g350900, Cre01.g048400*, and *Cre08.g381900*) (Table S4, Fig. 4B). Moreover, an uncharacterized gene (*Cre07.g357500*) containing a negative elongation factor A (*NELF-A*) domain was associated with *GPD2* (Fig. S11). Expression analysis confirmed that *bZIP* (*Cre16.g692250*) and *C2C2* (*Cre03.g146267*) were differentially expressed and their expression profile closely matched that of *GPD2* (Fig. 5B), suggesting their potential regulatory role under salinity stress.

In contrast, the TFs identified in the black module (*GPD3*) were members of the *SBP*, *MYB*-related, and *AP2* TF families, containing five, four, and three genes, respectively (Fig. 5C). Among the genes connected to *GPD3*, *Cre07.g320950* encodes a protein with both a CPG-binding protein domain and a PHD finger domain (*Alfin*-like 4) (Table S4, Fig. 5D). Although most of the genes connected to *GPD3* were uncharacterized, domain-based annotation revealed transcription regulatory functions, such as ankyrin repeat-containing domain in *Cre08.g365500*, a WD40 repeat-like domain in *Cre09.g397586*, and a NELF-A domain in *Cre10.g454350* (Fig. S13). These TFs expression profiles showed gradual up-regulation over time under NaCl treatment, with WD40, NELF-A and Ankyrin genes peaking expression between 8 h and 24 h, similar to *GPD3* (Fig. 5D).

Comparative analysis of the *GPD2* and *GPD3* promoter regions revealed less than 40% of sequence similarity (Fig. S17), indicating substantial divergence in their regulatory architecture. *Cis*-regulatory elements (CREs) analysis showed that both promoters share 16 CRE types, and three and eleven were unique for *GPD2* and *GPD3*, respectively (Table S6). Both promoters contain abscisic acid (ABA)-responsive elements (ABRE, ABRE3a, ABRE4) (Table S6), consistent with regulation by bZIP TFs (Wang et al., 2018; Guo et al., 2024). A GATA motif is also present in both promoter regions, however only *GPD2* is linked to an up-regulated *C2C2_GATA* TF (*Cre03.g146267*). Additionally, several GC-rich Sp1 motifs and zinc finger TFs in the magenta module point to additional regulatory inputs. In contrast, the *GPD3* gene promoter is enriched in MYC motifs recognized by basic helix-loop-helix (*bHLH*) TFs, which are found in the black module, and contains a GTGGAG motif corresponding to a known *Alfin*-like TF, consistent with the co-expressed gene *ALFIN*-like (*Cre07.g320950*) (Fig. 5C, Table S6).

These results indicate that shared stress-responsive elements coexist with gene-specific CREs, supporting a model in which *GPD2* and *GPD3* are regulated by distinct, temporally coordinated transcriptional programs during salinity response.

### Identification of lipid pathway genes in *GPD2*- and *GPD3*-associated modules

The magenta and black modules were screened for co-expressed genes involved in lipid metabolism, particularly those related to glycerol and triacylglycerol biosynthesis. In the magenta module (*GPD2*), 12 lipid-related genes were identified including sphingolipid, glycerophospholipid, and lipid metabolism pathways (Table S7). Among them, five lipases and triacylglycerol lipases (*Cre05.g248200, Cre06.g275150, Cre10.g425100, Cre10.g463600, Cre17.g699100*), and a gene encoding a phosphatidate phosphatase (*Cre12.g506600*) that catalyzes the conversion of phosphatidic acid to diacylglycerol were identified, (Deng, Cai & Fei, 2013) (Fig. S18). The black module (*GPD3*) contained 19 lipid-related genes including glycerophospholipid, sphingolipid, fatty acid biosynthesis, and glycerolipid metabolism (Table S7). Notably, a plastidic lysophosphatidic acid acyltransferase (*LPAAT; Cre09.g398289*), which catalyzes the conversion of LPA to phosphatidic acid (PA), was identified (Fig. S18). Additionally, several lipases and triacylglycerol lipases including *TGL7* (*Cre03.g174950*), *CPL19* (*Cre04.g219200*), *Cre10.g422850*, and *LIP2* (*Cre12.g498750*) and enzymes involved in fatty acid biosynthesis such as a 3-ketoacyl-CoA synthase (*Cre07.g319600*) and an acetyl-CoA biotin carboxyl carrier protein (*Cre08.g373050*) were identified (Fig. S18). These findings highlight that *GPD2* and *GPD3* are co-expressed with distinct sets of genes involved in the synthesis and recycling of precursors for glycerol and triacylglycerol biosynthesis during the salinity stress response.

### Validation of module assignments using machine learning

To evaluate the internal consistency of gene-to-module assignments obtained through WGCNA, we implemented a Random Forest classifier using normalized gene expression profiles (15,323 genes × 22 samples). The dataset was divided into training (12,270 genes; 80%) and test sets (3,053 genes; 20%), with upsampling to address class imbalance across 28 modules. The classifier achieved strong performance (cross-validated accuracy = 95.4%; Kappa = 0.95), and test-set evaluation maintained robust accuracy (70.9%; Kappa = 0.685; global AUC = 0.949) (Fig. S19). These results indicate that the modules represent well-separated expression patterns that can be independently recognized by a supervised classifier.

Performance analysis revealed strong classification metrics for the modules magenta and black, containing *GPD2* and *GPD3*, respectively. The magenta module achieved a balanced accuracy (BA) of 0.79, with high specificity (0.99) and moderate sensitivity (0.60), while the black module showed a higher BA of 0.86, with sensitivity of 0.74 and a positive predictive value of 0.73. Both modules exhibited excellent discriminative performance, with AUC values of 0.97 (magenta) and 0.98 (black) (Table S8). Across the network, the highest class-specific accuracies were observed for the turquoise (BA = 0.888), green (BA = 0.884), and pink (BA = 0.874) modules, whereas smaller modules showed lower performance, as expected (Fig. 2B, Table S7). Importantly, these results were obtained on a held-out test set, genes not used during model training, demonstrating that an independent algorithm can correctly assign genes to the same modules defined by WGCNA based solely on their expression profiles. UMAP projections and confusion matrices further confirmed that well-defined modules form distinct clusters, with misclassifications occurring primarily near module boundaries (Figs. S20–S21).

## Discussion

Glycerol biosynthesis is a central component of osmotic responses, and *GPD* genes are essential for glycerol-3-phosphate production during salt stress responses across photosynthetic organisms, including *C. reinhardtii* (Zhao et al., 2019; Zhao et al., 2021; Hang et al., 2020). In previous studies have demonstrated that *GPD2* and *GPD3* are particularly NaCl-responsive among the five *GPD* gene family in *C. reinhardtii* (Herrera-Valencia et al., 2012; Casais-Molina et al., 2016; Morales-Sánchez et al., 2017). However, the regulatory mechanisms underlying their coordinated yet distinct induction remained largely unexplored.

Here, we reanalyzed publicly available time-course RNA-seq data from *C. reinhardtii* exposed to 200 mM NaCl (Zhang et al., 2022). Our analysis confirmed that *GPD2* and *GPD3* are significantly up-regulated in response to salinity condition (Fig. 1), in agreement with previous studies (Casais-Molina et al., 2016; Morales-Sánchez et al., 2017); and provides new evidence that, despite their high sequence similarity and shared metabolic function (Fig. 1A), the genes *GPD2* and *GPD3* operate within distinct regulatory programs that are temporally organized during salinity stress (Fig. 1B, Fig. S5).

Zhang et al. (2022) reported a time-dependent transcriptional shift in *C. reinhardtii* under salinity stress. In line with this, our data highlight 8 h as a transcriptional inflection point, marked by the highest number of up-regulated genes (Fig. S4) and revealing that *GPD2* as an early-response gene, whereas *GPD3* is associated with later-responding genes consistent with roles in long-term adaptation (Fig. 1B, Fig. S5). This temporal partitioning aligns with previously reported critical window (6–8 h) for metabolic reconfiguration under abiotic stress (Boyle et al., 2012; Atikij et al., 2019), suggesting that glycerol pathway regulation is not only quantitatively controlled but also finely tuned in time.

The modular organization of the co-expression network further supports this interpretation (Fig. 3), suggesting different regulatory mechanisms or functional contexts during salinity response, while both genes share a common role in glycerol biosynthesis (Herrera-Valencia et al., 2012). This is consistent with functional studies showing condition-specific divergence; for instance, Driver et al. (2017) demonstrated that *GPD3* is overexpressed under phosphorus deficiency disrupted glycerolipid metabolism and reduced growth, chlorophyll, and glycerol levels, whereas *GPD2* overexpression had no major effects. Similarly, complementation of *GPD*-deficient *S. cerevisiae* with *GPD2* enhanced glycerol production and NaCl tolerance compared to *GPD3* (Casais-Molina et al., 2016). Additionally, *GPD2* exhibits substantially higher connectivity than *GPD3* (37 *vs.* 14 direct links), despite being located in a smaller module (447 genes in magenta module *vs.* 1,130 genes in black module), indicating its integration into a dense and highly coordinated early-response network when transcriptional reprogramming is most active. Conversely, *GPD3* show fewer but functionally coherent associations consistent with specialized roles during prolonged stress and response maintenance (Zhang et al., 2022; Vilarrasa-Blasi et al., 2022; Farkas et al., 2023) (Fig. 4, Figs. S10–S13, Table S4).

Notability, genes co-expressed with *GPD2* and *GPD3* include key salinity stress responders. *GPD2* is associated with a Calmodulin-Binding Protein-like gene (*Cre06.g270750*), similar to Arabidopsis *CML13/14* proteins that interact with calmodulin-binding activators to maintain osmotic balance (Hau et al., 2024). In contrast, *GPD3* connects to an up-regulated Na^+^-dependent cotransporter (*Cre02.g095085*), critical for ion homeostasis, and to uncharacterized genes with the cytoskeletal remodeling domain calponin homology (*Cre12.g489450*) and WASP-interacting protein (*Cre13.g605900*), linked to osmotic stress adaptation (Wang, Zhang & Huang, 2011; Palani et al., 2021) (Fig. S13, Table S4).

Also, hub gene analysis revealed that magenta and black modules are driven by salinity-responsive genes (Fig. 4). In the magenta module, *P-type ATPase* (*Cre09.g410050*) and cysteine dioxygenase 1 (*CDO1*) were identified as top hubs (Fig. 4A). *P-type ATPases* are involved in active transport of cations such as Na^+^ and Ca^2^^+^ across biological membranes (Palmgren, 2023). *CDO1* co-expression in this early-response module is notable given that reactive oxygen species (ROS) accumulate during salt stress in *C. reinhardtii* (Shetty, Gitau & Maróti, 2019), and cysteine dioxygenases in plants have been shown to function in ROS detoxification and as oxygen sensors regulating ERF-VII transcription factor degradation (Taylor-Kearney & Flashman, 2022). In the black module, hub genes include *OGT1* and *PP2C* (Fig. 4C). *GPD3* co-expressed with *OGT1*, an N-acetylglucosaminyltransferase involved in O-glycosylation linked to stress responses (Mathieu-Rivet et al., 2020), which is particularly relevant given the post-translational regulation of *GPD2 via* phosphorylation under salinity (Cruz-Powell et al., 2024). *OGT* genes are also salt-responsive in Arabidopsis, where their deficiency disrupts cytokinin signaling (Yoo et al., 2021). *PP2C* regulates osmotic stress through the ABA signaling pathway by modulating AREB/ABF transcription factors (Jung, Nguyen & Cheong, 2020). Moreover, *GPD2* and *GPD3* co-expressed with key genes involved in glycerol and TAG production (Fig. S18), well-characterized responses to salt stress in *C. reinhardtii* (Merchant et al., 2012; Park et al., 2015; Hang et al., 2020).

Integration of TFs co-expression with *cis*-regulatory element analyses indicates that *GPD2* is preferentially associated with early stress-responsive TFs, such as *bZIP* (*Cre16.g692250)*, *C2C2_GATA (Cre03.g146267)*, and *APRR1*-like (Fig. 5). The binding domains were identified in *GPD2* gene promoter region (ABRE, As-1, G-box, and TGA-box) and whose conserved roles in salt stress signaling have been well established in different plants, including rice and grapevine (Geniza et al., 2023; Zhang et al., 2023; Guo et al., 2024) (Fig. 5B, Table S6). In contrast, *GPD3* is linked to TFs including *ALFIN*-like, *bHLH*, *SBP*, and *MYB*-related proteins (Fig. 5D), together with corresponding *cis*-regulatory elements, suggesting regulation tailored to later adaptive phases. These results are in line with previous studies showing the TFs up-regulation, such as *ALFIN*-like in wheat plants under salt stress (Liu et al., 2024). Additionally, the identification of a WD40 repeat-like domain in an uncharacterized gene (*Cre09.g397586*) co-expressed with *GPD3* suggests potential regulatory functions relevant to salinity response (Fig. S13). WD40 proteins have been extensively studied in salt stress responses: the GbLWD1-like WD40 TF enhances salt tolerance in transgenic *A. thaliana* (Xin et al., 2021), and OsABT, a rice WD40 protein, increases salt tolerance in *Arabidopsis* by interacting with ABA signaling components (Eryong & Bo, 2022).

**Figure 6:**
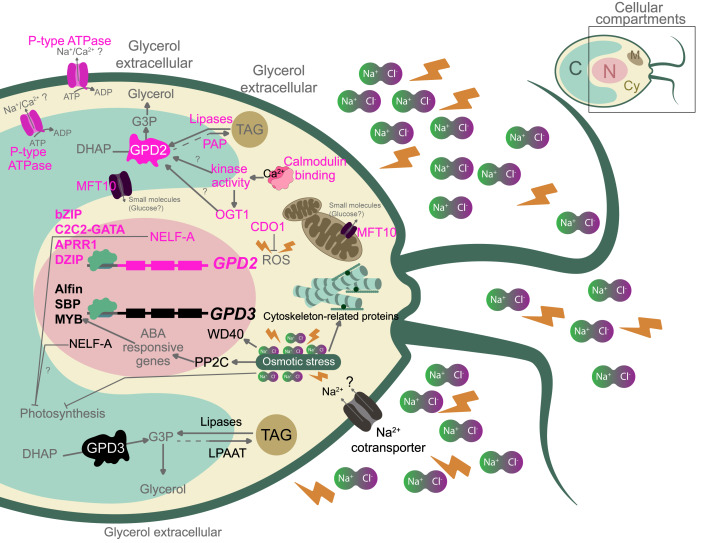
Model of *C. reinhardtii* response associated with the *GPD2* and *GPD3* networks under salt stress. The diagram summarizes *GPD2* and *GPD3* associations with transcription factors and molecular components involved in the salinity response. Cellular compartments: nucleus (N), chloroplast (C), cytosol (Cy), mitochondria (M). Transcription factors and regulatory proteins: Alfin, Arabidopsis Pseudo Response Regulator 1 (APRR1), Basic Leucine Zipper (bZIP), C2C2 Zinc Finger GATA (C2C2-GATA), DZIP family ring finger protein (DZIP), myeloblastosis-like (MYB), Negative Elongation Factor A (NELF-A), Squamosa Promoter Binding Protein (SBP), WD40 repeat domain (WD40). Enzymes and metabolites: Cysteine Dioxygenase 1 (CDO1), Dihydroxyacetone Phosphate (DHAP), Glycerol-3-Phosphate (G3P), Lysophosphatidic Acid Acyltransferase (LPAAT), Protein Phosphatase 2C (PP2C), Triacylglycerol (TAG). Other components: Cation-transporting ATPase (P-type ATPase), Major Facilitator Superfamily Transporter 10 (MFT10), and Reactive Oxygen Species (ROS).

Based on these results, we constructed schematic model of the *C. reinhardtii* response to salinity stress, centered on the *GPD2* and *GPD3* co-expression network, revealing distinct regulatory and functional contexts (Fig. 6). The model highlights associations with transcription factors and key genes involved in glycerol and TAG biosynthesis, along with conserved domains such as *NELF-A* (Fig. 6), which, although not previously reported in plants, may contribute to transcriptional repression of stress-sensitive pathways (Yamaguchi et al., 2002; Diao, Su & Vos, 2025).

From a methodological perspective, our study introduces a complementary strategy that integrates unsupervised network inference with supervised machine learning. The Random Forest classifier (RF) has been used for high-dimensional transcriptomic data due to its robustness and ability to detect complex expression patterns (Ghosh & Cabrera, 2022). Unlike previous studies that combine RF and WGCNA for biomarker discovery (Lv et al., 2023; Li et al., 2024), in our study, RF was used as an internal validation tool to assess the separability and robustness of WGCNA-derived gene modules. The strong classification performance and high discriminative power (global AUC = 0.949), particularly for modules containing *GPD2* and *GPD3* genes (Figs. S19–S21, Table S8), supports the structural coherence of the network and strengthens confidence in the biological interpretability of the regulatory programs identified.

The robustness of our results is further supported by multiple validation steps. First, we demonstrated that the observed responses are reproducible across experimental context by overlapping with external RNA-seq dataset expose to salinity reported by Vilarrasa-Blasi et al. (2022). Similar validation strategies have been used in different transcriptomic studies (Bouquet et al., 2016; Wang et al., 2020). Second, we demonstrate concordance (*r* > 0.90) by including qPCR-validated genes reported by Zhang et al. (2022). Placing experimentally supported stress-responsive genes within the co-expression network provides an independent biological reference to assess module coherence, regulatory context, and network connectivity. Together, these results enhance the reliability of our findings and the biological and functional relevance of the inferred networks.

Finally, this work highlights the scientific value of publicly available transcriptomic resources. It has become an increasingly powerful strategy for scientific discovery, enabling insights from conserved developmental programs or stress-responsive networks in land plants (Julca et al., 2021; Soto-Cardinault, Childs & Góngora-Castillo, 2023) to the reconstruction of transcriptional regulatory circuits in microalgae under abiotic stress (López García De Lomana et al., 2015). By applying an integrative analytical framework to existing RNA-seq data, we identified regulatory features that were not apparent in the original analyses. Overall, our findings advance the understanding of salinity responses in green microalgae and provide a conceptual and analytical framework for identifying regulatory targets with potential relevance for future efforts to enhance salt tolerance in *C. reinhardtii* and related photosynthetic systems.

## Conclusions

This study provides new insights into the transcriptional regulation within the *GPD* gene family that shapes glycerol biosynthesis under salinity stress in *C. reinhardtii*. We provide evidence that *GPD2* and *GPD3* could be controlled by distinct transcriptional programs despite their functional similarity. This level of regulatory resolution could be particularly relevant for biotechnological strategies aimed at enhancing salt stress tolerance in microalgae.

The integration of co-expression analysis with supervised machine learning provides a robust framework for identifying and prioritizing regulatory components in complex transcriptional landscapes. This approach is widely applicable to stress-resistant algae strains and provides a transferable framework for analyzing regulatory networks in other biological systems, helping to guide future efforts in functional genomics and metabolic optimization.

##  Supplemental Information

10.7717/peerj.21060/supp-1 Supplemental Information 1 Supplemental figures and tables

10.7717/peerj.21060/supp-2 Supplemental Information 2 Normalized gene expression with the variance stabilization transformation (VST) function under salinity condition used for the WGCNA analysis

10.7717/peerj.21060/supp-3 Supplemental Information 3 Results of the differential expression analysis comparing treatment time points under salinity conditions with the untreated control

## Additional Information and Declarations

### Data Availability

The following information was supplied regarding data availability: The code used in this study is available at Github and Zenodo: – https://github.com/jorgetzec/WGCNA_ML_validation
– Tzec-Interián & Góngora-Castillo (2026). jorgetzec/WGCNA_ML_validation: WGCNA_ML_validation –v1.1 (v1.1). Zenodo. https://doi.org/10.5281/zenodo.18393687. The RNA sequencing data are available at the Short Read Archive (SRA): PRJNA770825.
