# Supplementary material for: Integrating co-expression network analysis and machine learning to reveal the regulatory landscape of GPD genes in Chlamydomonas reinhardtii under salinity stress
Source: PeerJ. 2026 Apr 14;14:e21060. doi: 10.7717/peerj.21060 (PMC13089224; doi:10.7717/peerj.21060)
Supplement: Supplemental Information 1 [file peerj-14-21060-s001.pdf]

Integrating co-expression network analysis and machine learning to reveal the regulatory landscape of *GPD* genes in *Chlamydomonas reinhardtii* under salinity stress

Jorge A. Tzec-Interián[1], Santy Peraza-Echeverria[1], Virginia Aurora Herrera-Valencia[1],* and Elsa B. Góngora-Castillo[2]*

[1] Unidad de Biotecnología, Centro de Investigación Científica de Yucatán, Mérida, Yucatán, México
[2] Secihti-Departamento de Recursos del Mar, Centro de Investigación y de Estudios Avanzados del Instituto Politécnico Nacional, Mérida, Yucatán. México

Corresponding Author:

Elsa B. Góngora-Castillo[2]

Email address: elsa.gongora@cinvestav.mx

Virginia Aurora Herrera-Valencia[1]

Email address: vicky@cicy.mx

Table S1. Number of reads processed of *C. reinhardtii* transcriptome under NaCl 200 mM and reads mapped to *C. reinhardtii* genome.

Time	Sample	Total processed reads	Reads aligned (%)	Reads assigned (%)
0 h	0h_1	64,565,134	92.8	85
	0h_2	57,051,752	91.5	83.2
	0h_3	48,720,822	93	84.6
2 h	2h_1	58,833,816	92.5	88.2
	2h_2	74,599,804	92.	83
	2h_3	54,934,298	91.5	85.4
4 h	4h_1	62,276,286	92.3	85.5
	4h_2	67,923,934	91.8	84.9
	4h_3	66,830,934	91.6	82.7
8 h	8h_1	58,110,462	92.7	84.5
	8h_2	60,749,986	92	84.1
	8h_3	47,640,920	92.2	84
12 h	12h_1	57,397,562	91.7	85.2
	12h_2	54,520,628	91.3	85
	12h_3	66,026,050	91.4	85
24 h	24h_1	52,497,330	90.3	84.7
	24h_2	57,328,362	89.3	83.9
	24h_3	55,270,834	89.2	84.1
48 h	48h_1	60,818,302	90.7	84.5
	48h_2	63,372,090	87.5	81.6
	48h_3	60,260,368	88.7	82.6
72 h	72h_1	54,017,246	79.7	76.6
	72h_2	53,570,258	84.6	80
	72h_3	59,168,744	86.4	81

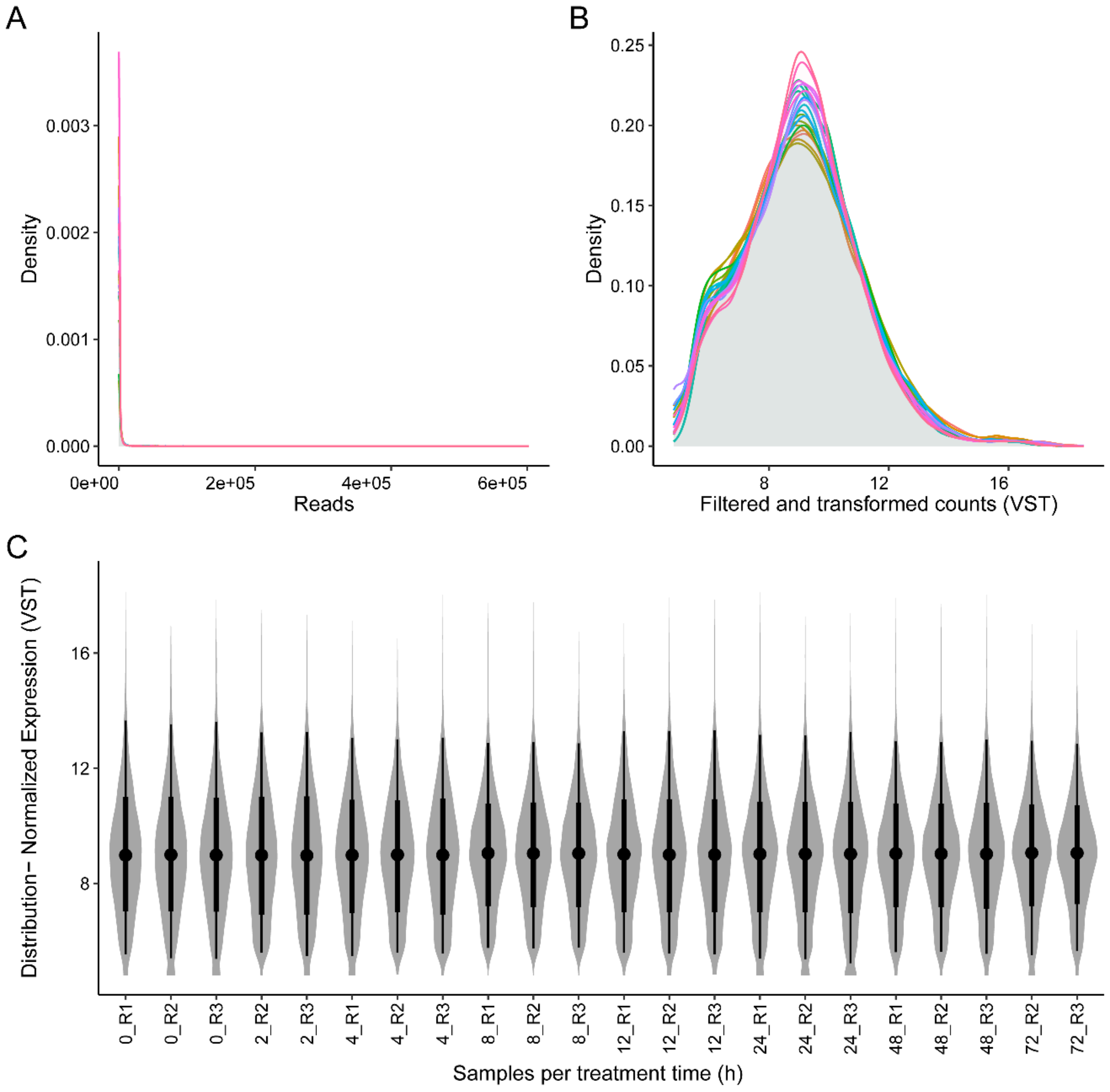

Figure S1. Density of gene expression data before and after filtering and normalization with the variance stabilization transformation (VST) function. (A) Density of expression values from raw counts for each of the 24 samples and 17,741 genes analyzed. (B) Density of expression values filtered and normalized with VST for 22 samples and 15,323 genes. (C) Data distribution of the 22 samples after filtering and normalization with the VST function. 0_R1 refers to control without NaCl treatment (0 h), replicate 1.

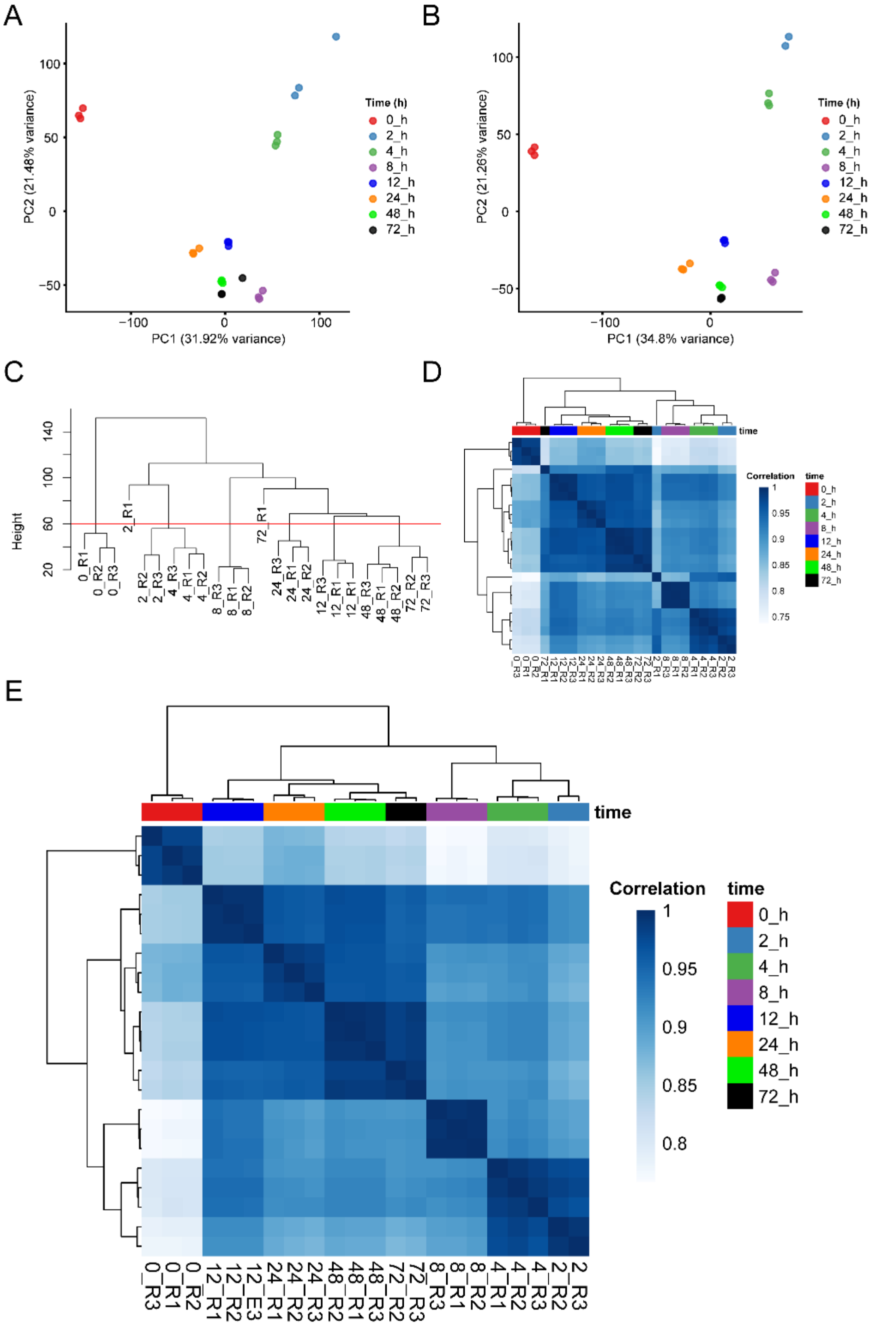

Figure S2. Sample outlier detection. (A) Principal component analysis (PCA) of the 24 original samples (0–72 h) on VST-normalized expression values, showing overall grouping by time point prior to outlier removal. (B) PCA of the dataset after removing two outlier samples (2 h replicate 1 and 72 h replicate 1), showing improved within-group consistency. (C) Hierarchical clustering of the 24 samples using Euclidean distances and the average linkage method on VST-normalized expression values. The red line indicates the height threshold used to define outliers. (D) Pearson pairwise correlation heatmap for all 24 samples before outlier removal, highlighting reduced correlation of the two excluded samples with their biological replicates. (E) Pearson's correlation heatmap after outlier removal, showing increased sample coherence across the remaining 22 samples containing 15,323 genes.

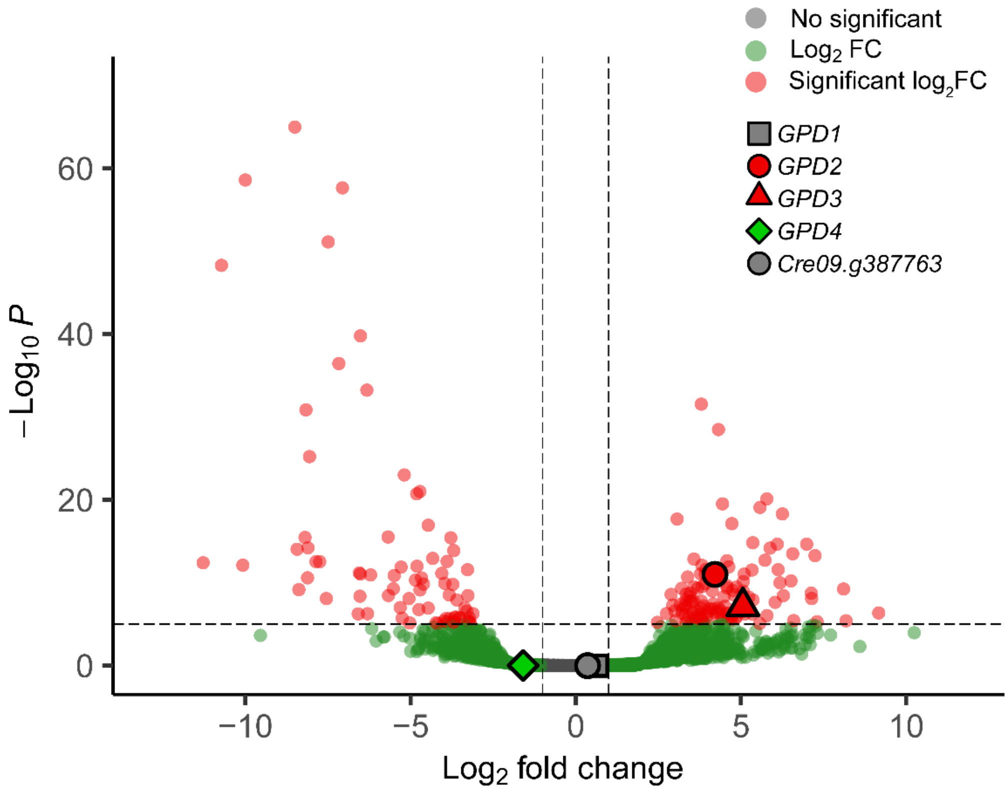

Figure S3. Volcano plot of differentially expressed *GPDs* genes. A total of 15323 genes is represented with 252 significant up-regulated and 136 significant down-regulated genes at $Log_2FC = 2$ and $p.adj < 0.05$.

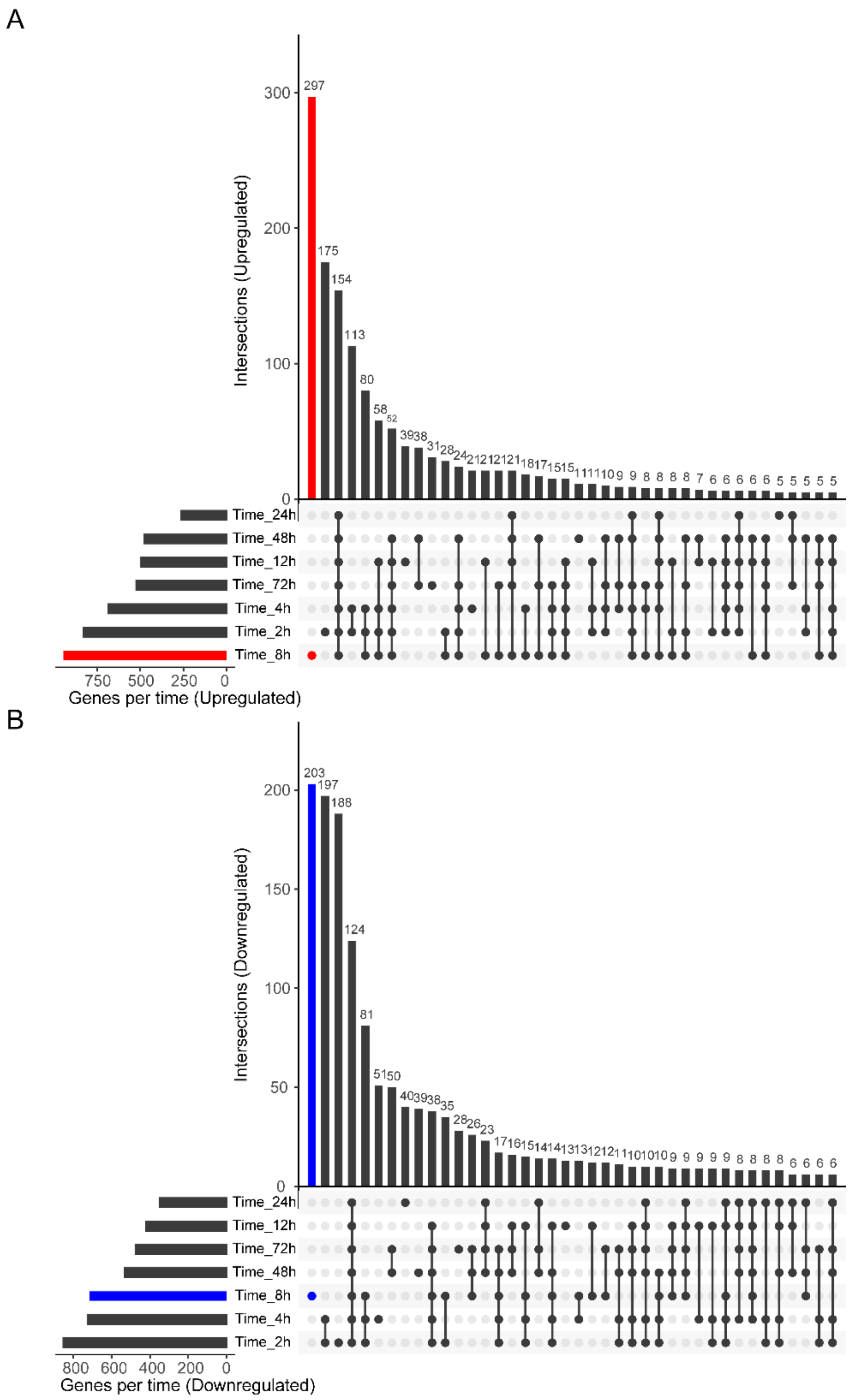

Figure S4. Differential gene expression analysis in response to treatment with 200 mM NaCl at different time points. (A) Intersections of up-regulated genes with a threshold of Log$_2$ fold change (Log2FC) > 2 and p-adj < 0.05. Treatment time of 8 h includes the highest and unique number of up-regulated genes and is colored red. (B) Intersections of down-regulated genes with the same threshold. Treatment time of 2, 4, and 8 h includes the highest and unique number of down-regulated genes and is colored blue. The total number of genes analyzed was 15323. Unique up- and down-regulated genes were identified between the different treatment times.

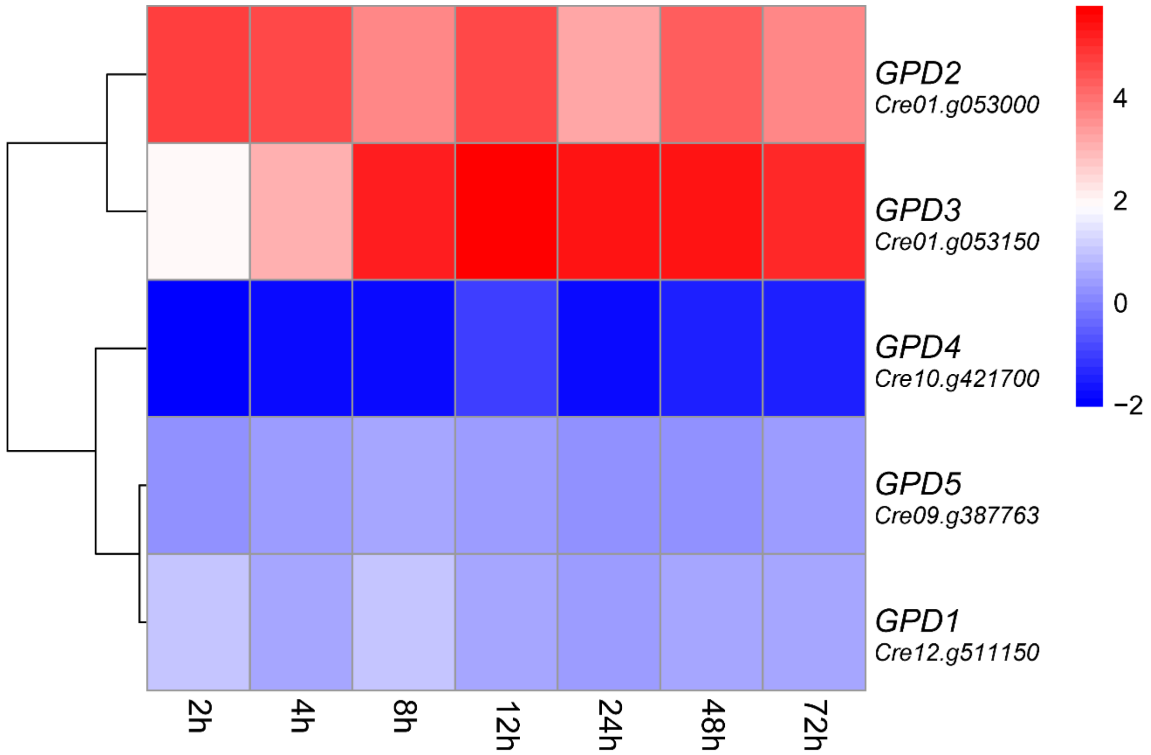

Figure S5. Differential expression of *GPD* genes over time under salinity conditions. The heatmap displays the log_2 fold change (log_2FC) values obtained from differential expression analysis when treatments are compared to controls at different time-points, with a significance threshold of $Log_2FC \geq 2$ and adjusted p-value (p.adj) < 0.05.

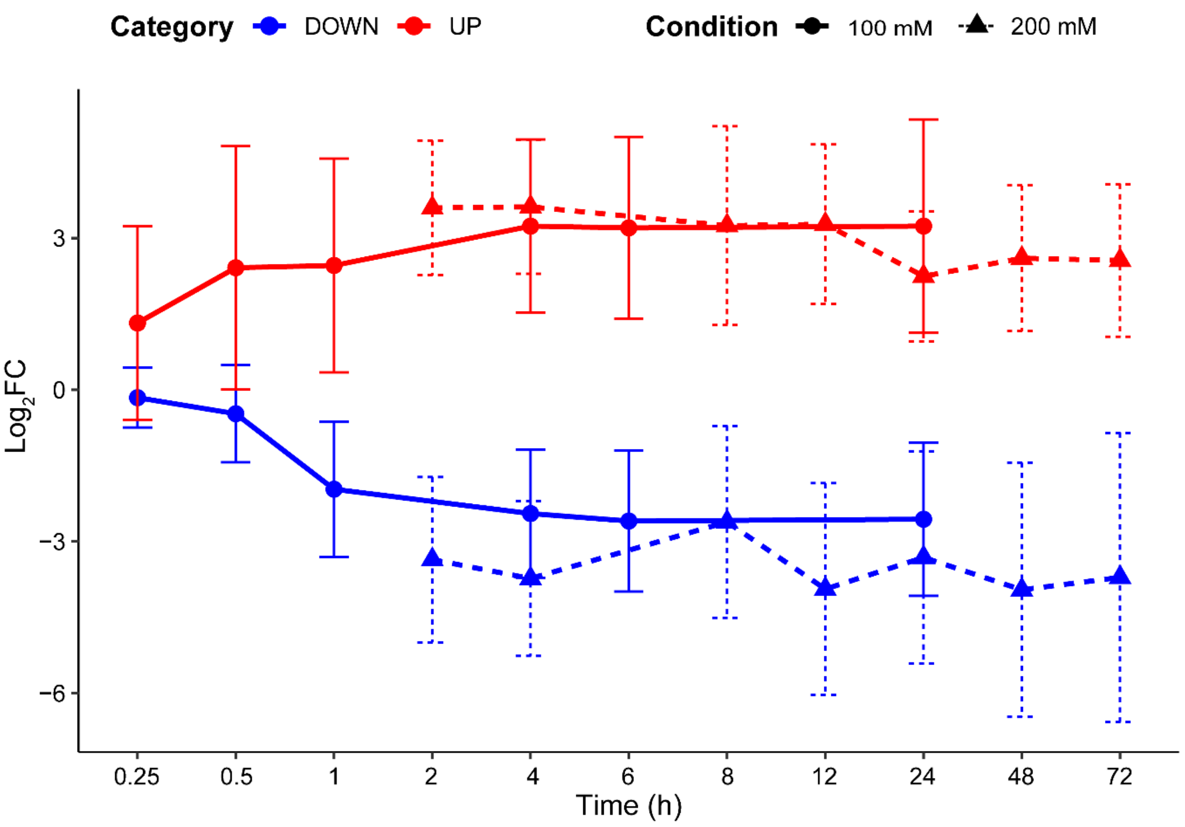

Figure S6. Expression profiles of differentially expressed genes consistently regulated under salinity stress in two independent RNA-seq datasets. A total of 40 genes were consistently up-regulated and 63 down-regulated at either 4 h or 24 h in both datasets: a published experiment using 100 mM NaCl (0.25 h, 0.5 h, 1 h, 4 h, 6 h, 24 h), and this study using 200 mM NaCl (2 h, 4 h, 8 h, 12 h, 24 h, 48 h, 72 h). Each line represents the mean $\log_2$ fold change (± standard deviation) across time points for the shared gene set.

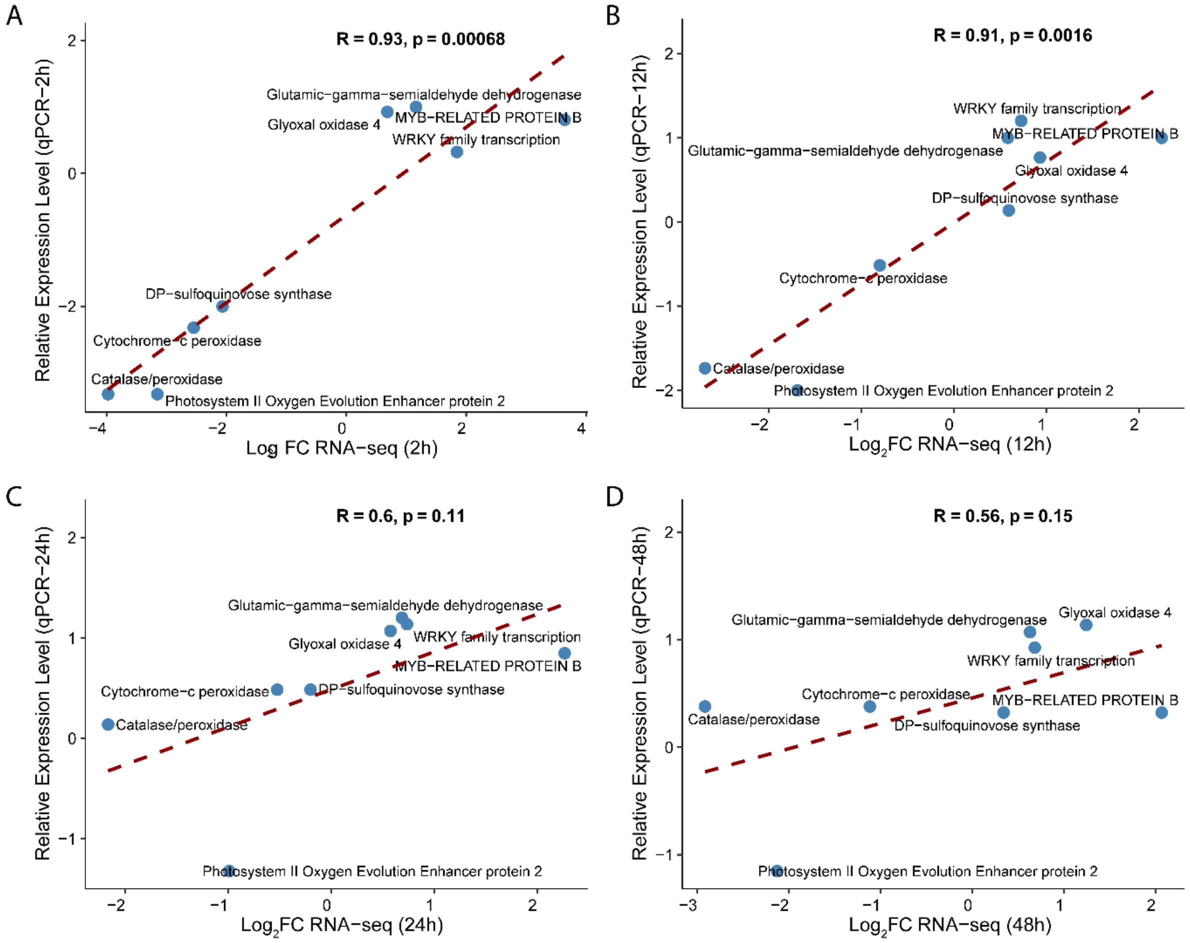

Figure S7. Correlation between RNA-seq and qPCR-derived expression changes at different time points under 200 mM NaCl treatment. Scatter plots show the relationship between RNA-seq-derived Log_2 fold change (Log_2FC) and qPCR mean relative expression values for eight representative genes. (A) Correlation at 2 h. (B) Correlation at 12 h. (C) Correlation at 24 h. (D) Correlation at 48 h.

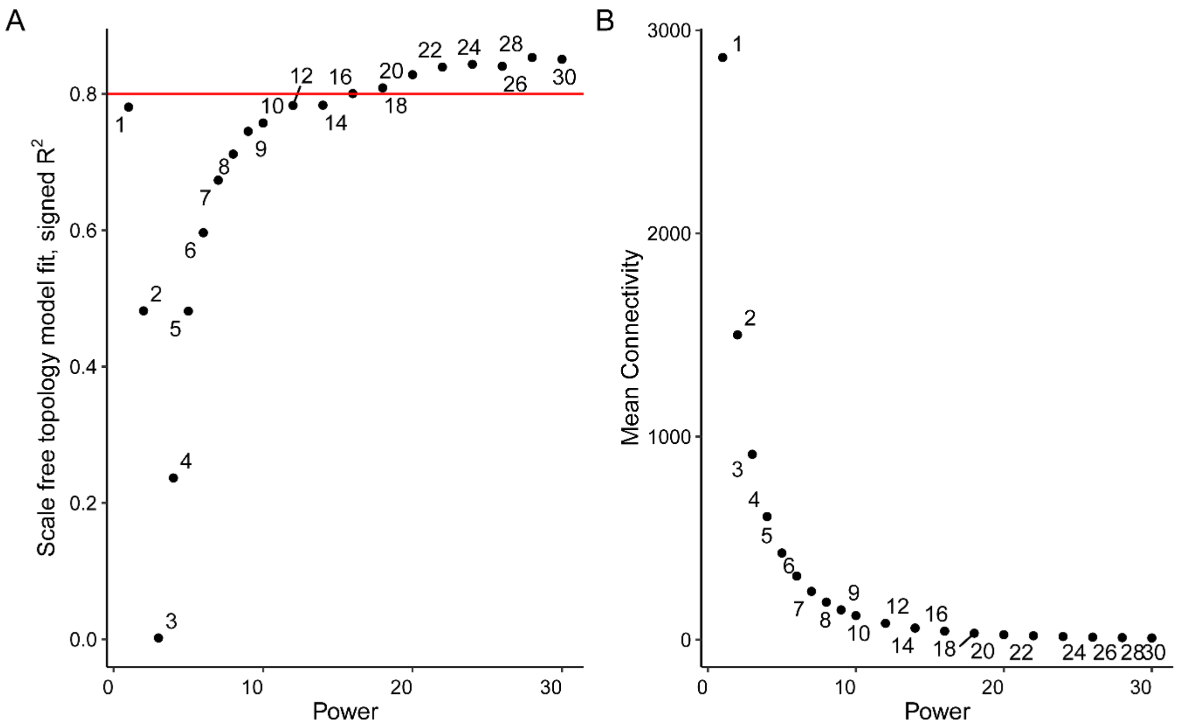

Figure S8. Power and mean connectivity analysis for co-expression network analysis. (A) Power analysis. (B) Mean connectivity analysis.

Table S2. Number of differentially expressed genes in modules identified from the comparison of samples treated with 200 mM NaCl versus untreated controls, with a threshold of log2foldchange >2 and padj. <0.05.

**Module**	**Up-regulated**	**Down-regulated**
black	19	0
blue	27	0
green	0	107
greenyellow	1	0
magenta	18	0
midnightblue	2	0
pink	0	11
purple	2	0
turquoise	183	0
yellow	0	21

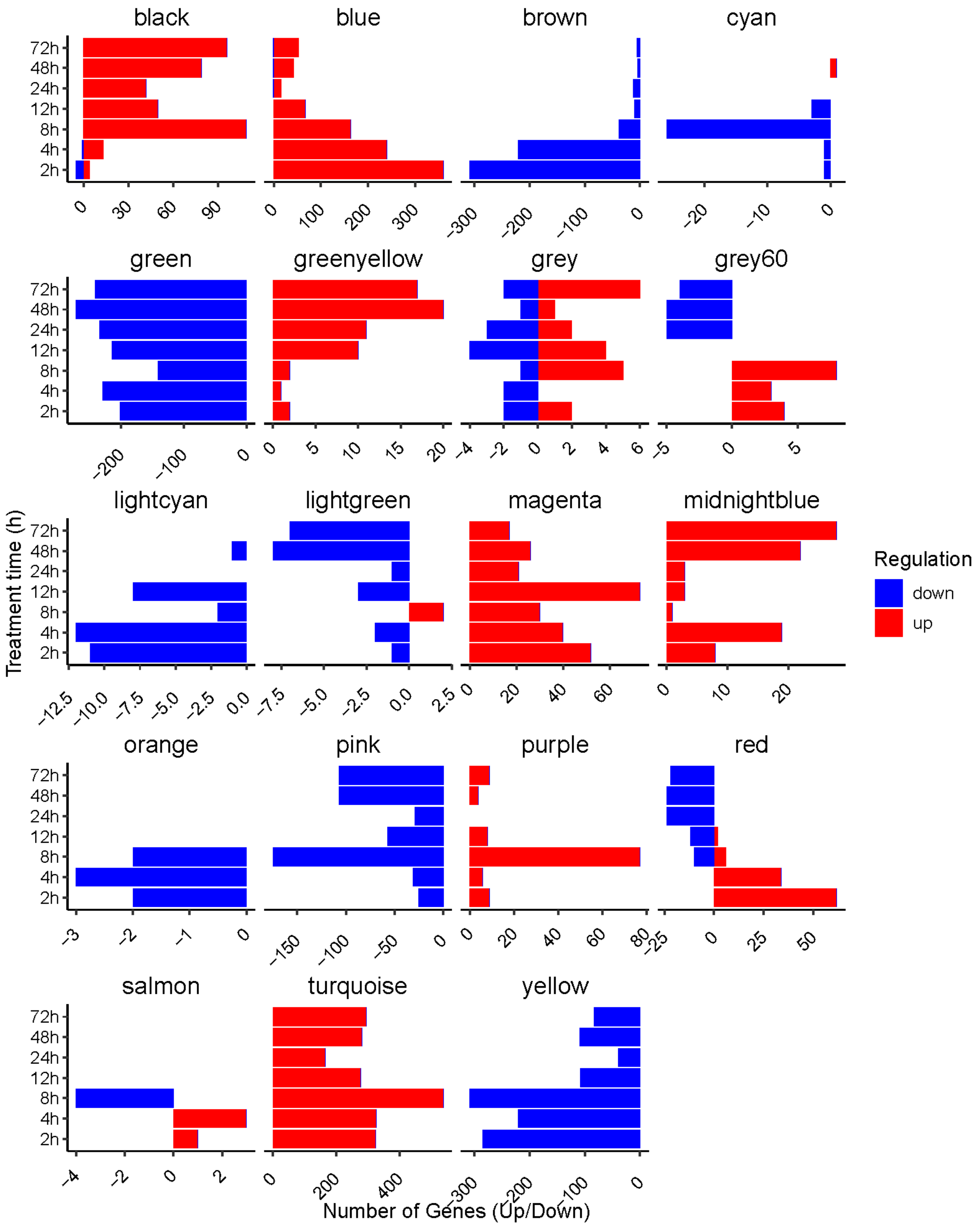

Figure S9. Genes differentially expressed at each treatment time with 200 mM NaCl in each module of the gene co-expression network of the C. reinhardtii transcriptome. Genes were identified at a threshold of log2foldchange>2 and padj<0.05. Modules "darkgreen", "darkgrey", "darkred", "lightyellow", "royalblue", "tan", "white", and "darkorange" were omitted as having fewer than 3 differentially expressed genes at only 1 or 2 treatment times.

Table S3. Significant molecular functions (MF) and biological processes (BP) enriched in the co-expression modules (adjusted p-value < 0.05).

Module	ONT	GO_ID	Term	Sig.	Exp.	Weightfisher	p-Adj
turquoise	MF	GO:0003676	nucleic acid binding	150	104.43	4.50E-05	0.0016
turquoise	MF	GO:0008270	zinc ion binding	30	14.38	6.30E-05	0.0016
turquoise	MF	GO:0003677	DNA binding	66	44.39	0.00067	0.0112
turquoise	MF	GO:0016779	nucleotidyltransferase activity	9	2.31	0.00332	0.0415
turquoise	BP	GO:0009451	RNA modification	16	3.98	1.70E-05	8.00E-04
turquoise	BP	GO:0043414	macromolecule methylation	7	1.16	3.70E-05	9.00E-04
turquoise	BP	GO:0009190	cyclic nucleotide biosynthetic process	23	11.16	0.00036	0.006
turquoise	BP	GO:0001522	pseudouridine synthesis	8	2.52	0.00223	0.0279
blue	MF	GO:0004672	protein kinase activity	99	63.11	1.70E-06	1.00E-04
blue	MF	GO:0000166	nucleotide binding	71	53.87	0.0006	0.015
blue	MF	GO:0003677	DNA binding	59	41.13	0.0025	0.0417
blue	BP	GO:0000398	mRNA splicing, via spliceosome	10	1.64	3.40E-07	0
brown	MF	GO:0003735	structural constituent of ribosome	28	12.16	2.00E-05	0.001
brown	BP	GO:0008152	metabolic process	85	70.88	2.70E-06	1.00E-04
brown	BP	GO:0034645	cellular macromolecule biosynthetic proc...	8	1.64	8.40E-05	0.0021
brown	BP	GO:1901566	organonitrogen compound biosynthetic pro...	13	4.49	0.00025	0.0042
brown	BP	GO:1901137	carbohydrate derivative biosynthetic pro...	5	1.04	0.00216	0.027
brown	BP	GO:0009058	biosynthetic process	26	21.95	0.00463	0.0435
brown	BP	GO:0044271	cellular nitrogen compound biosynthetic ...	15	16.34	0.00522	0.0435
yellow	MF	GO:0010181	FMN binding	6	1.23	0.00079	0.0212
yellow	MF	GO:0003824	catalytic activity	248	203.37	0.00085	0.0212
yellow	BP	GO:0009765	photosynthesis, light harvesting	16	2.19	3.80E-12	0
yellow	BP	GO:0008152	metabolic process	94	74.69	0.00035	0.0087
green	MF	GO:0015079	potassium ion transmembrane transporter ...	5	1.01	9.50E-05	0.0048
green	MF	GO:0005315	inorganic phosphate transmembrane transp...	5	0.81	0.00071	0.0178

red	MF	GO:0004175	endopeptidase activity	25	9.69	6.50E-10	0
red	MF	GO:0005198	structural molecule activity	16	15.53	3.80E-06	1.00E-04
red	MF	GO:0016758	hexosyltransferase activity	6	1.33	0.0014	0.02
red	MF	GO:0005515	protein binding	119	93.35	0.0016	0.02
red	BP	GO:0006886	intracellular protein transport	8	1.11	3.30E-06	2.00E-04
red	BP	GO:0006810	transport	26	9.2	6.90E-05	0.0017
red	BP	GO:0033365	protein localization to organelle	5	0.69	0.00027	0.0045
red	BP	GO:0030163	protein catabolic process	4	0.69	0.00328	0.041
pink	MF	GO:0004129	cytochrome-c oxidase activity	6	0.67	1.40E-05	7.00E-04
pink	MF	GO:0016491	oxidoreductase activity	50	22.06	0.00052	0.013
magenta	MF	GO:0000166	nucleotide binding	27	17.74	0.00043	0.0215
magenta	BP	GO:0008652	cellular amino acid biosynthetic process	4	0.42	0.00057	0.0243
magenta	BP	GO:0016311	dephosphorylation	4	0.49	0.00097	0.0243
purple	MF	GO:0005215	transporter activity	8	5.58	0.00085	0.0425
greenyellow	BP	GO:0042309	homoiothermy	15	6.1	0.00014	0.007
lightcyan	MF	GO:0005488	binding	57	42.95	0.00036	0.0068
lightcyan	MF	GO:0005515	protein binding	29	14.78	0.0004	0.0068
lightcyan	MF	GO:0016776	phosphotransferase activity, phosphate g...	3	0.15	0.00041	0.0068
lightcyan	MF	GO:0019205	nucleobase-containing compound kinase ac...	3	0.18	0.00064	0.008
grey60	BP	GO:0009190	cyclic nucleotide biosynthetic process	6	0.99	0.00019	0.0095
darkgrey	MF	GO:0016627	oxidoreductase activity, acting on the C...	2	0.04	0.00058	0.029

ONT: Ontology GO;_ID: Unique identifier for the Gene Ontology term; Sig.: Number of genes in the module associated with the GO term; Exp.: Number of genes expected to be associated with the GO term under a random distribution; weightFisher: Raw p-value obtained using the "weight01" algorithm and Fisher's exact test; p-adj: Adjusted p-value using the Benjamini & Hochberg method.

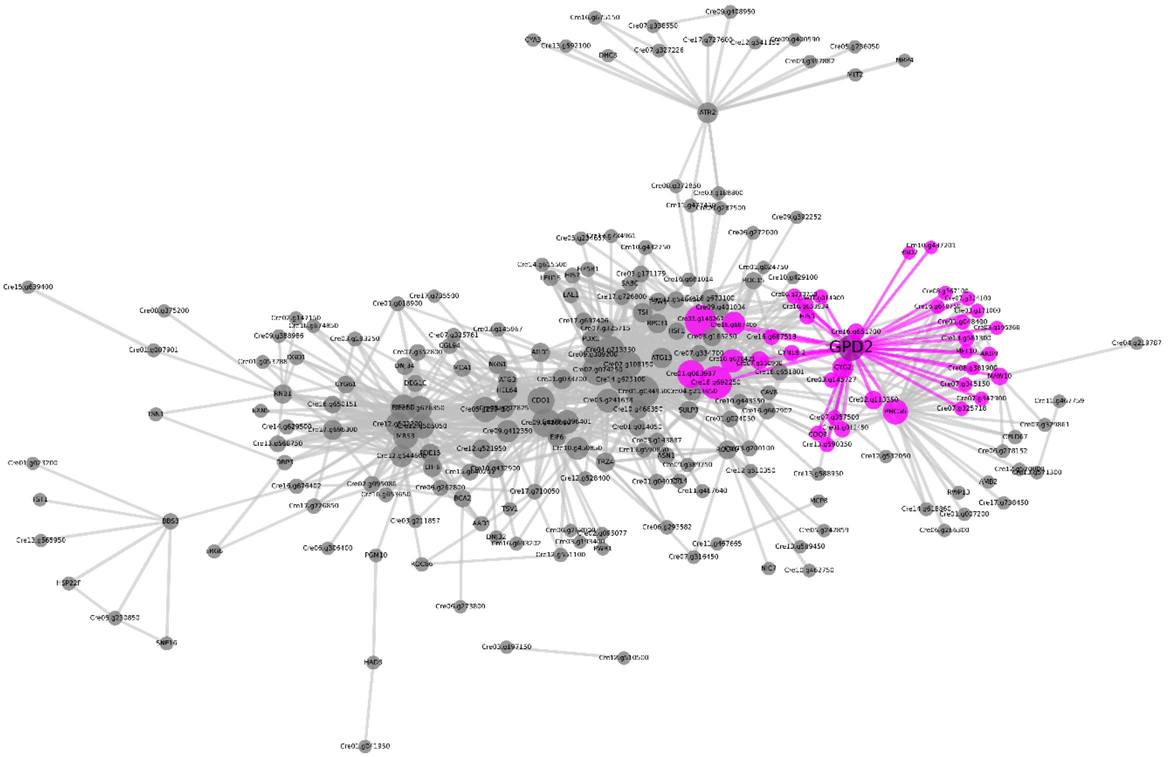

Figure S10. Magenta module in gene co-expression network for *C. reinhardtii* transcriptome under 200 mM NaCl treatment. Network was constructed with a weight threshold of 0.17. The location of *GPD2* within the network is indicated, with its 15 most significant connections highlighted in magenta.

Table S4. Expressed genes connected with *GPD2* and *GPD3* in the network co-expression analysis for the *C. reinhardtii* transcriptome under 200 mM NaCl treatment. Genes within the magenta and black modules that are connected to *GPD2* and *GPD3* genes, ranked by node degree (number of connections).

Module	Gene ID	Description	Node Degree	Log_2FC
Magenta	*Cre16.g692250*	Basic-leucine zipper (bZIP) transcription factor	66	3.39*
	Cre03.g146267	C2C2_GATA transcription factor (Ankyrin repeats (Ank_4))	61	6.26*
	Cre01.g063997	ATAXIN 2-RELATED (PTHR12854)	43	1.34
	GPD2 (Cre01.g053000)	Glycerol-3-phosphate dehydrogenase/dihydroxyacetone-3-phosphate reductase	37	4.22*
	PHC56 (Cre16.g654600)	Pherophorin-chlamydomonas homolog 56	34	2.71*
	Cre16.g687406	Unknown	27	3.94*
	Cre16.g676421	Two-Component Response Regulator-Like APRR1-Related (PTHR26402:SF465)	27	6.99*
	CYG2 (Cre06.g259500)	Adenylate/guanylate cyclase	20	5.88*
	Cre07.g350900	Ankyrin repeats (Ank_2) // Zinc finger, C3HC4 type (RING finger) (PF12796//PF13920)	12	1.74
	Cre02.g113350	Protein tyrosine kinase	12	0.81
	MAW10 (Cre07.g325760)	Membrane-associated hydroxyproline-rich glycoprotein 10	10	2.23
	Cre16.g651700	Unknown	9	2.13
	CYN18-2 (Cre12.g499400)	Cyclophilin	7	1.57
	Cre16.g653934	Unknown	7	1.89
	Cre13.g581300	Unknown	6	1.28
	Cre01.g012450	E3 Ubiquitin-Protein Ligase HUWE1 (PTHR11254:SF291)	6	1.76
	Cre16.g687518	EIF2B1-Translation initiation factor eIF-2B subunit alpha (K03239)	6	1.57
	HIS5 (Cre16.g672385)	Histidinol phosphate aminotransferase	5	1.16
	Cre03.g145727	Tyrosine-TRNA Ligase (PTHR11946:SF53)	5	1.35
	Cre06.g270750	Calmodulin binding protein-like (PF07887)	5	1.10
	COQ9	Ubiquinone biosynthesis protein	5	1.45

	ARG9 (Cre06.g278163)	Acetylornithine aminotransferase	4	1.42
	Cre13.g590350	bZIP transcription factor	4	0.93
	Cre01.g014900	Unknown	4	0.67
	Cre07.g357500	Unknown	4	0.88
	Cre01.g048400	zinc finger protein DZIP1 (K16470)	4	1.96
	MFT10 (Cre02.g095076)	Major facilitator superfamily transporter	3	3.38*
	Cre07.g325718	Unknown	3	4.8*
	Cre08.g367100	Unknown	3	1.98
	Cre03.g171000	Non-specific serine/threonine protein kinase / Threonine-specific protein kinase (2.7.11.1)	3	1.12
	Cre07.g324100	Dopamine Beta Hydroxylase Related (PTHR10157)	3	0.93
	Cre07.g345150	Alpha-tubulin N-acetyltransferase / Tubulin N-acetyltransferase (2.3.1.108)	3	1.15
	Cre07.g347900	F-BOX And WD40 Domain Protein (PTHR22844)	3	1.50
	Cre16.g648750	SMG-7 Suppressor with Morphological Effect On Genitalia Protein 7 (PTHR15696)	3	1.05
	Cre03.g195366	Unknown	2	3.08*
	Cre08.g381900	Ring Zinc Finger Protein (PTHR22763)	2	0.62
	BSD2 (Cre16.g678773)	3-beta hydroxysteroid dehydrogenase	1	0.74
	Cre10.g437201	Serine-Threonine Protein Kinase (PTHR23257)	1	1.28
Black	*Cre10.g454350*	Unknown	94	1.84
	OGT1 (Cre12.g552851)	*O*-linked *N*-acetylglucosamine transferase	90	3.85*[+]
	Cre17.g741272	Unknown	40	2.72
	Cre03.g201650	Unknown	35	2.46
	Cre08.g358600	Unknown	16	2.08
	GPD3 (Cre01.g053150)	Glycerol-3-phosphate dehydrogenase/dihydroxyacetone-3-phosphate reductase	14	5.06*
	Cre11.g481150	Unknown	12	2.89
	Cre08.g365500	Unknown	6	1.23
	Cre12.g489450	Unknown	5	1.61
	Cre09.g397586	Unknown	4	1.44
	Cre13.g605900	Unknown	3	1.14
	Cre02.g095084	Glycosyltransferase 14 family member (PTHR19297)	2	4.83*

Cre07.g320950	CPG Binding Protein // PHD Finger Protein Alfin-Like 4 (PTHR12321//PTHR12321:SF55)	2	0.74
Cre08.g380800	Unknown	2	1.39
Cre02.g095085	Predicted Na+-dependent cotransporter (KOG4821)	1	4.62*

Significance (*p.adj* < 0.05) at the Log2FC value is indicated with asterisks (*).
Hub genes are indicated with + symbol.

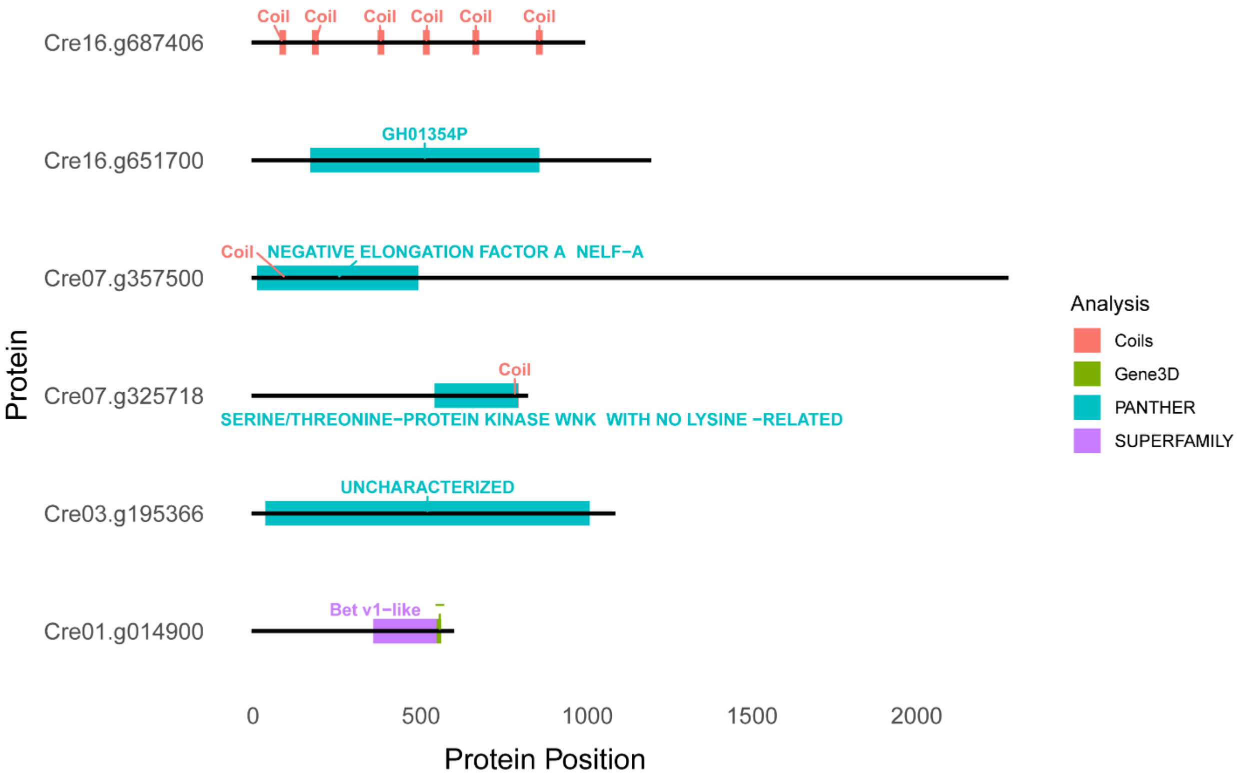

Figure S11. Functional annotation of proteins encoded by uncharacterized genes connected to *GPD2* in the magenta module. Annotation was performed using InterProScan (v5.59-91.0). Black lines represent the length of the proteins, illustrating the domains and functional motifs identified with bars.

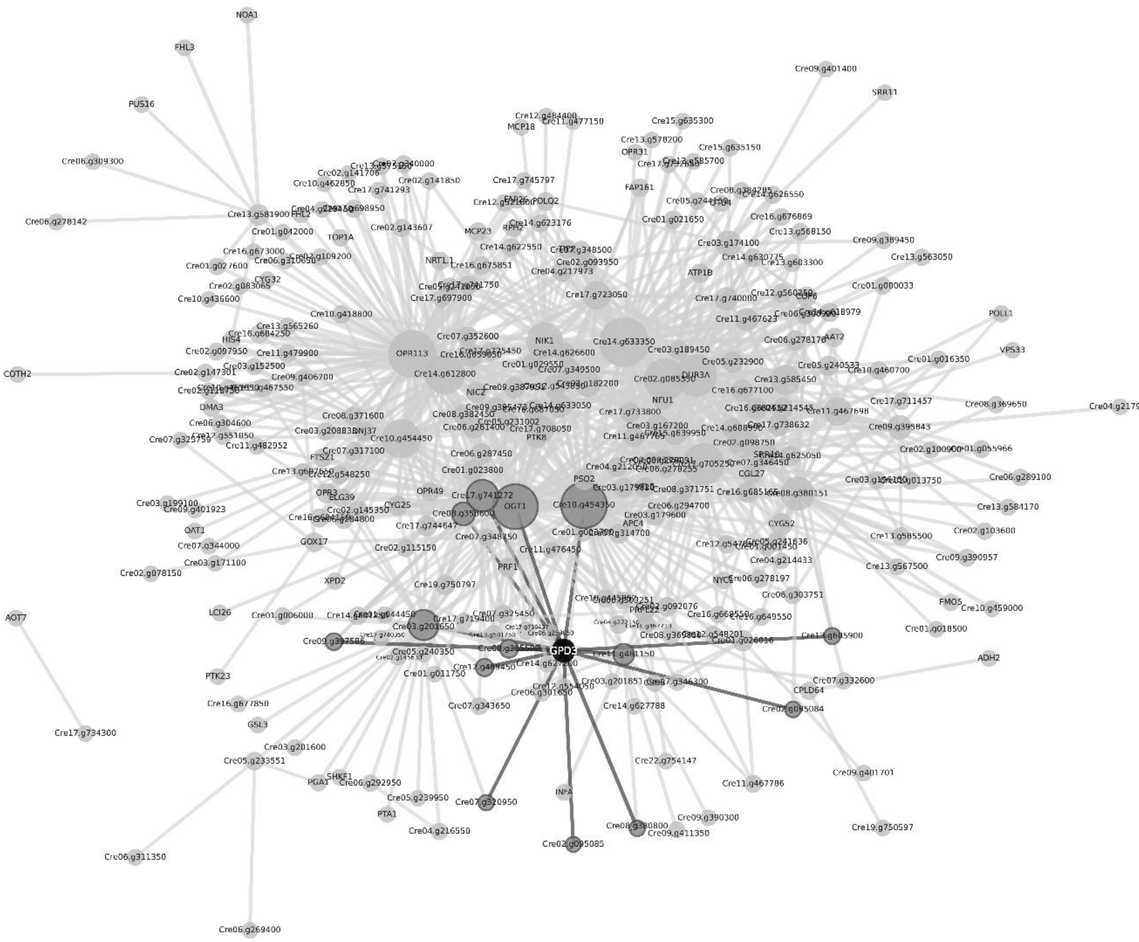

Figure S12. Black module in gene co-expression network for *C. reinhardtii* transcriptome under 200 mM NaCl treatment. Network was constructed with a weight threshold of 0.17. The location of *GPD3* within the network is indicated into the black circle with white text, with its connections highlighted in gray circles and black text.

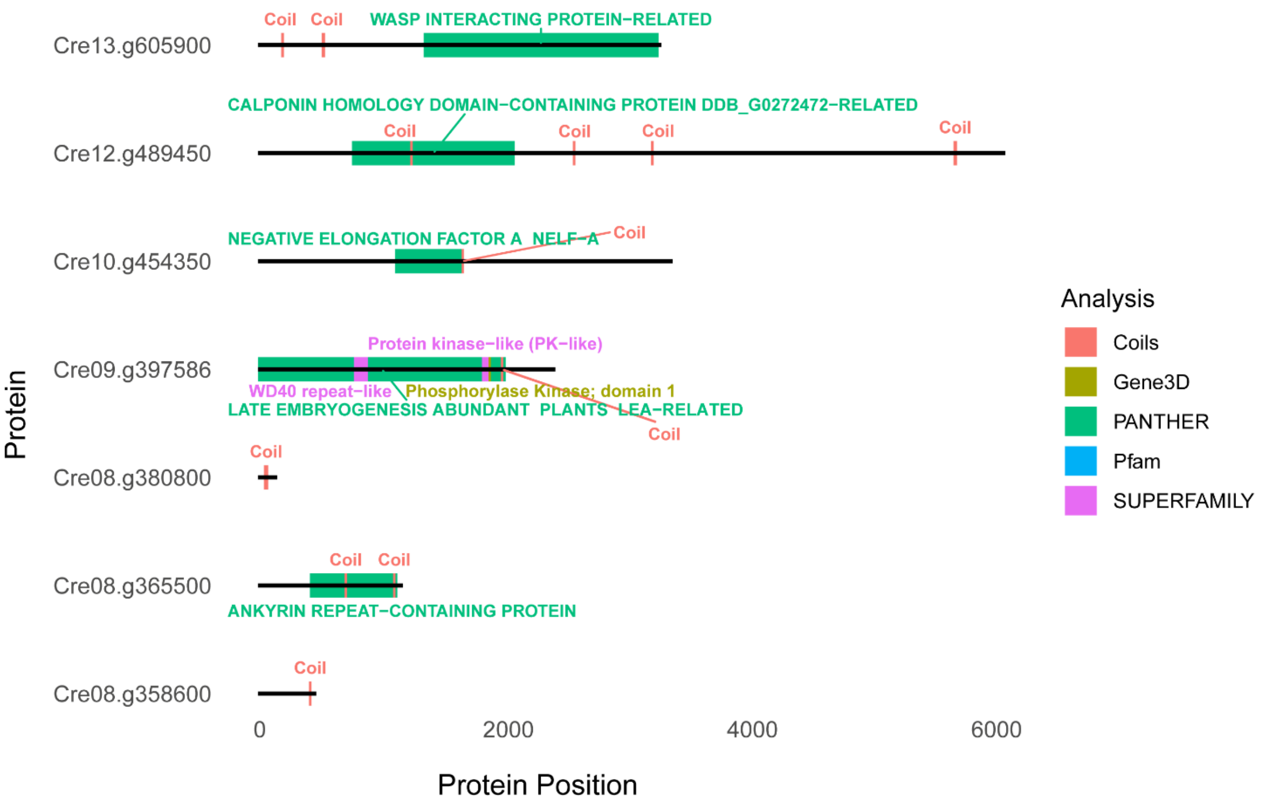

Figure S13. Functional annotation of proteins encoded by uncharacterized genes connected to *GPD3* in the black module. Annotation was performed using InterProScan (v5.59-91.0). Black lines represent the length of the proteins, illustrating the domains and functional motifs identified with bars.

Table S5. Node degree (Connectivity) of qPCR-validated genes across weight thresholds in co-expression modules.

**Gene**	**Module**	**Annotation**	**0.02**	**0.05**	**0.1**	**0.15**	**0.17**	**0.20**
**Cre17.g732350**	black	Glyoxal oxidase 4	290	134	21	2	0	0
**Cre02.g103450**	blue	MYB-RELATED PROTEIN B	1210	977	603	315	210	60
**Cre04.g228400**	blue	WRKY family transcription	1291	991	511	127	49	22
**Cre03.g146527**	salmon	Glutamic-gamma-semialdehyde dehydrogenase	139	90	19	2	0	0

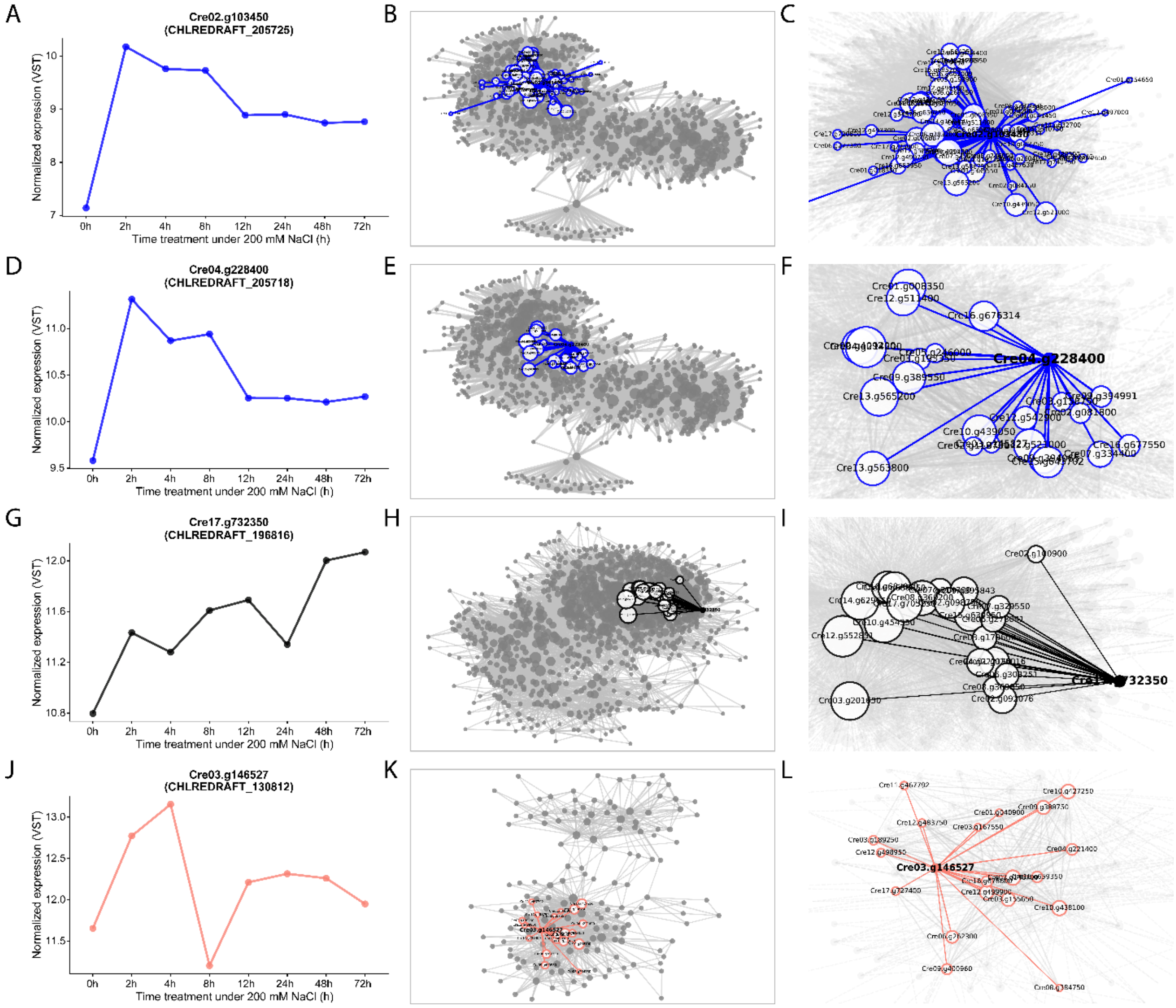

Figure S14. Expression profiles, co-expression subnetworks, and close-up views of gene neighborhoods validated by qPCR under 200 mM NaCl in *C. reinhardtii*. (A) Expression profile of *MYB* (*Cre02.g103450*). (B) MYB-centered network within the blue module (weight threshold = 0.20). (C) Subnetwork of MYB. (D) Expression profile of *WRKY* TF (*Cre04.g228400*). (E) WRKY-centered network within the blue module (weight threshold = 0.20). (F) Subnetwork of *WRKY*. (G) Expression profile of glyoxal oxidase 4 (*GLOX4*; *Cre17.g732350*). (H) *GLOX4*-centered network within the black module (weight threshold = 0.10). (I) Subnetwork of *GLOX4*. (J) Expression profile of glutamic-γ-semialdehyde dehydrogenase (*GSDH*; *Cre03.g146527*). (K) *GSDH*-centered network within the salmon module (weight threshold = 0.15). (L) Subnetwork of *GSDH*.

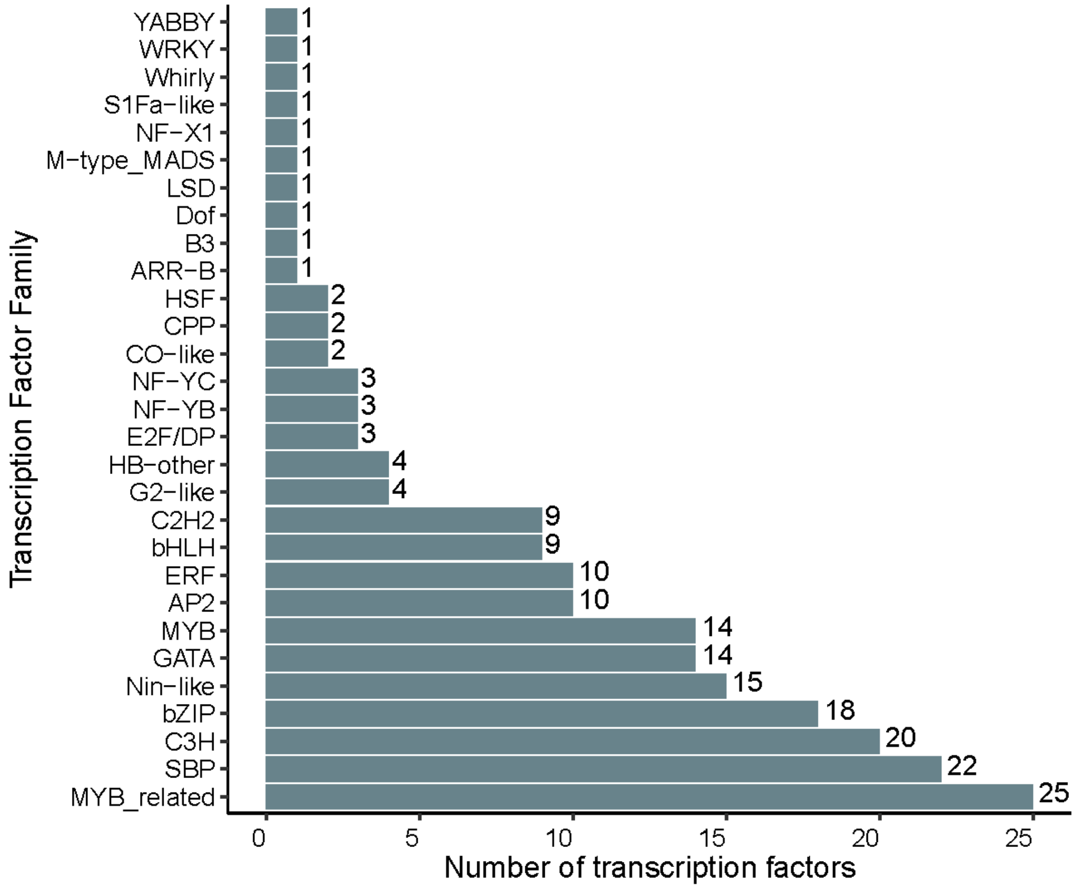

Figure S15. Number of transcription factors per family identified in the gene co-expression network of *C. reinhardtii* under salinity conditions at 200 mM NaCl.

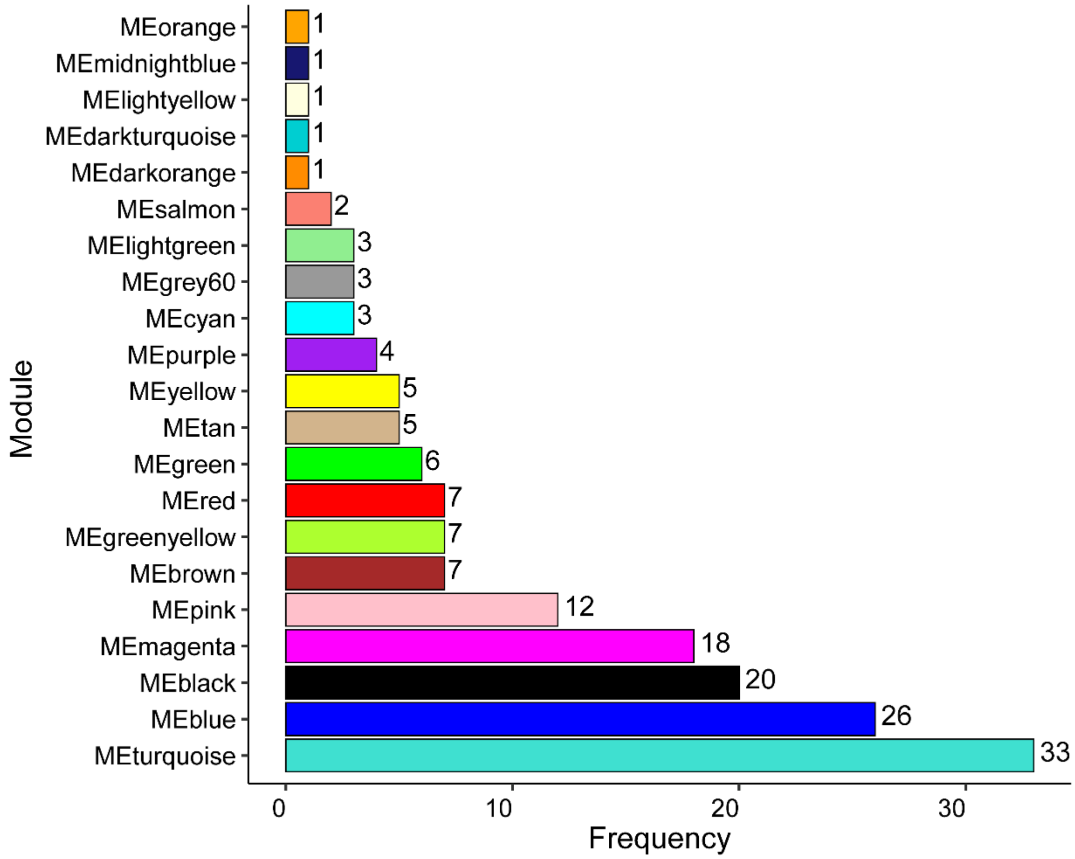

Figure S16. Number of transcription factors identified per module in the co-expression network of the *Chlamydomonas reinhardtii* transcriptome under 200 mM NaCl treatment. Transcription factors were annotated using PlantTFDB version 5 based on *C. reinhardtii* genome version 5.5.

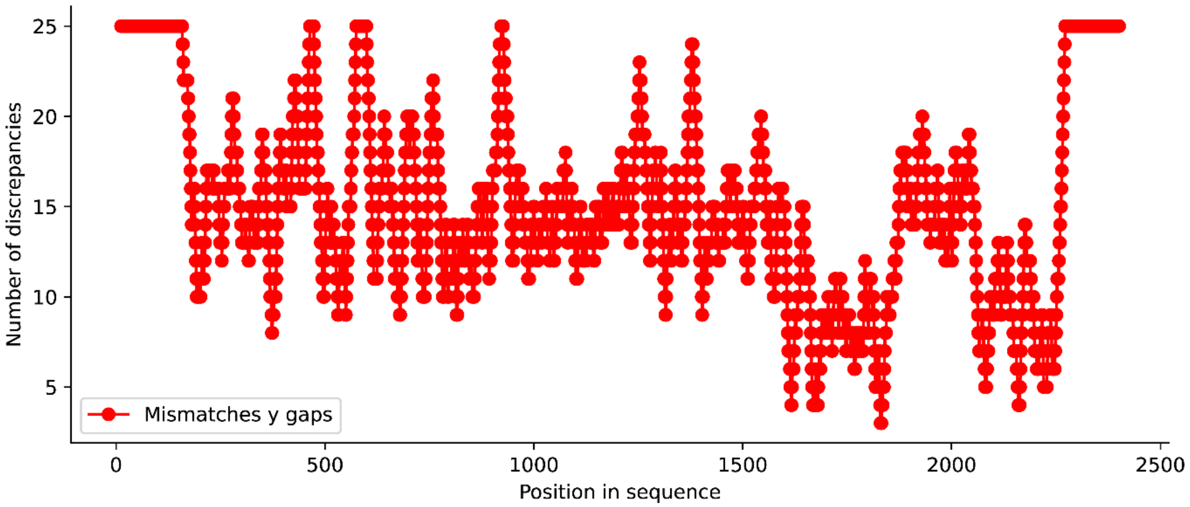

Figure S17. Discrepancy graph of the 2-kbp promoter regions of *GPD2* and *GPD3* genes aligned using the Clustal Omega algorithm (EMBL-EBI). The graph quantifies mismatches and gaps in 25-bp windows, illustrating sequence variation and conservation across these promoter regions.

Table S6. Number of *Cis*-regulatory elements (CREs) within the 2000 bp upstream promoter regions of *GPD2* and *GPD3* genes in *C. reinhardtii*.

CRE Type	*GPD2*	*GPD*3	Function
AAGAA-motif	0	1	--
A-box/ CCGTCC motif/ CCGTCC box	9	4	*cis*-acting regulatory element
ABRE/ ABRE3a/ ABRE4	5	15	*cis*-acting element involved in the abscisic acid responsiveness
AC-I	0	1	--
ACTCATCCT sequence	1	0	--
ARE	1	3	*cis*-acting regulatory element essential for the anaerobic induction
as-1/ TGACG-motif/ CGTCA-motif	9	5	*cis*-acting regulatory element involved in the MeJA-responsiveness
AT~ABRE	0	1	--
AT-rich element	0	1	binding site of AT-rich DNA binding protein (ATBP-1)
AuxRR-core	1	0	*cis*-acting regulatory element involved in auxin responsiveness
box S	0	2	
CAAT-box	20	20	common *cis*-acting element in promoter and enhancer regions, MYBHv1 binding site
CAT-box/ CCAAT-box	1	1	--
DRE core/ DRE1	3	1	--
GATA-motif	1	1	part of a light responsive element
G-box	3	6	*cis*-acting regulatory element involved in light responsiveness

GC-motif	3	1	enhancer-like element involved in anoxic specific inducibility
GT1-motif	0	2	light responsive element
LAMP-element	1	0	part of a light responsive element
LTR	1	1	*cis*-acting element involved in low-temperature responsiveness
MYB/ MYB recognition site/ Myb-binding site/ MYB-like sequence	5	6	MYB binding site involved in drought-inducibility
MYC	1	2	--
Sp1	3	1	light responsive element
STRE	7	9	--
TATA-box	0	4	core promoter element around -30 of transcription start
TCT-motif	0	1	part of a light responsive element
TGA-box	1	1	part of an auxin-responsive element
TGA-element	0	3	auxin-responsive element
W box	0	1	--
WRE3	0	2	--

Table S7. Genes involved in lipid metabolism in the magenta and black modules.

Module	Gene	Symbol	Phytozome Description/ AutoDefline	KEGG_ID	KEGG_gID	KEGG_path_desc
magenta	Cre01.g033450	--	(1 of 100) PTHR12393:SF6 - SPHINGOMYELIN PHOSPHODIESTERASE 2	600	CHLRE_01g033450v5	Sphingolipid metabolism
magenta	Cre01.g053000	GPD2	Glycerol-3-phosphate dehydrogenase/dihydroxyacetone-3-phosphate reductase	564	CHLRE_01g053000v5	Glycerophospholipid metabolism
magenta	Cre03.g198050	--	(1 of 3) PTHR19308 - Phosphatidylcholine Transfer Protein	--	--	--
magenta	Cre05.g248200	--	(1 of 2) PTHR21493//PTHR21493:SF124 - CGI-141-Related/Lipase Containing Protein // Subfamily Not Named	--	--	--
magenta	Cre06.g275150	CGL69	conserved protein with lipase motif	--	--	--
magenta	Cre09.g401034	--	(1 of 100) PTHR12393:SF6 - Sphingomyelin Phosphodiesterase 2	600	CHLRE_09g401034v5	Sphingolipid metabolism
magenta	Cre10.g425100	--	(1 of 1) PTHR24185//PTHR24185:SF1 - Family Not Named // Calcium-Independent Phospholipase A2-Gamma	--	--	--
magenta	Cre10.g450850	--	(1 of 1) PF00168//PF16016 - C2 domain (C2) // Domain of unknown function (DUF4782) (DUF4782)	--	--	--
magenta	Cre10.g463600	TGL17	Triacylglycerol lipase	--	--	--
magenta	Cre12.g506600	LPN1	Lipin family protein	565	CHLRE_12g506600v5	Ether lipid metabolism
magenta	Cre14.g618350	--	(1 of 100) PTHR12393:SF6 - Sphingomyelin Phosphodiesterase 2	600	CHLRE_14g618350v5	Sphingolipid metabolism
magenta	Cre17.g699100	TGL20	Triacylglycerol lipase	--	--	--
black	Cre01.g049750	--	(1 of 1) PTHR12714:SF11 - C-terminal s-isoprenylcysteine carboxyl o-methyltransferase	564	CHLRE_01g049750v5	Glycerophospholipid metabolism
black	Cre01.g053150	GPD3	Glycerol-3-phosphate dehydrogenase/dihydroxyacetone-3-phosphate reductase	564	CHLRE_01g053150v5	Glycerophospholipid metabolism

black	Cre02.g117200	--	Putative beta-galactosidase. GH42 family	600	CHLRE_02 g117200v5	Sphingolipid metabolism
black	Cre03.g174950	TGL7	Triacylglycerol lipase	--	--	--
black	Cre03.g182650	PGA1	Phospholipid/glycerol acyltransferase	--	--	--
black	Cre03.g183650	GDP4	Glycerophosphoryl diester phosphodiesterase family protein	--	--	--
black	Cre04.g218200	--	1 of 100) PTHR12393:SF6 - Sphingomyelin phosphodiesterase 2	600	CHLRE_04 g218200v5	Sphingolipid metabolism
black	Cre04.g219200	CPL19	possible serine esterase	--	--	--
black	Cre05.g244150	--	(1 of 100) PTHR12393:SF6 - SPHINGOMYELIN PHOSPHODIESTERASE 2	600	CHLRE_05 g244150v5	Sphingolipid metabolism
black	Cre07.g319600	FAE3	Putative 3-keto-acyl-CoA synthase	61	CHLRE_07 g319600v5	Fatty acid biosynthesis
black	Cre08.g373050	BCC3	Acetyl-CoA biotin carboxyl carrier	61	CHLRE_08 g373050v5	Fatty acid biosynthesis
black	Cre09.g397068	--	(1 of 100) PTHR12393:SF6 – Sphingomyelin phosphodiesterase 2	600	CHLRE_09 g397068v5	Sphingolipid metabolism
black	Cre09.g398289	LPAAT	related to plastidic lysophosphatidic acid acyltransferase (LPAAT)	561	CHLRE_09 g398289v5	Glycerolipid metabolism
black	Cre10.g422850		(1 of 5) PTHR11614:SF87 - Monoglyceride Lipase	--	--	--
black	Cre11.g467850	PGA6	Putative phospholipid/glycerol acyltransferase	--	--	--
black	Cre12.g498750	LIP2	Triacylglycerol lipase	--	--	--
black	Cre13.g585700	--	C2 domain (Calcium/lipid-binding domain, CaLB)	--	--	--
black	Cre15.g635300	--	(1 of 100) PTHR12393:SF6 - Sphingomyelin phosphodiesterase 2	600	CHLRE_15 g635300v5	Sphingolipid metabolism
black	Cre16.g671400	ARS1	Periplasmic arylsulfatase	600	CHLRE_16 g671400v5	Sphingolipid metabolism

KEGG_ID: KEGG database pathay ID; KEGG_gID: Gene ID in KEGG database; KEGG_path_desc: KEGG pathway description**.**

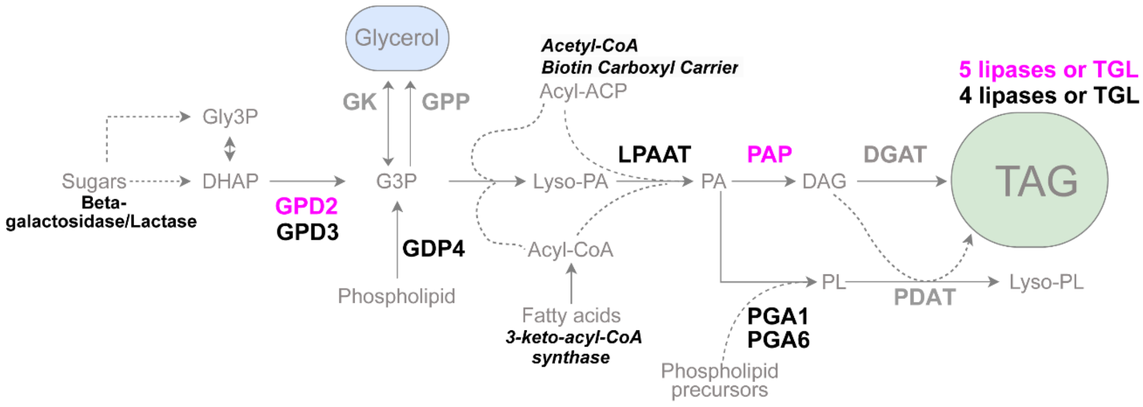

Figure S18. Metabolic pathway of glycerol and triacylglycerol (TAG) biosynthesis in *C. reinhardtii*, highlighting *GPD2* and *GPD3* participation and their co-expression associations under salinity. The diagram emphasizes *GPD2* and *GPD3* roles within glycerol and TAG metabolism. Genes co-expressed with *GPD2* and *GPD3* are highlighted in magenta and black, corresponding to the magenta and black modules, respectively.

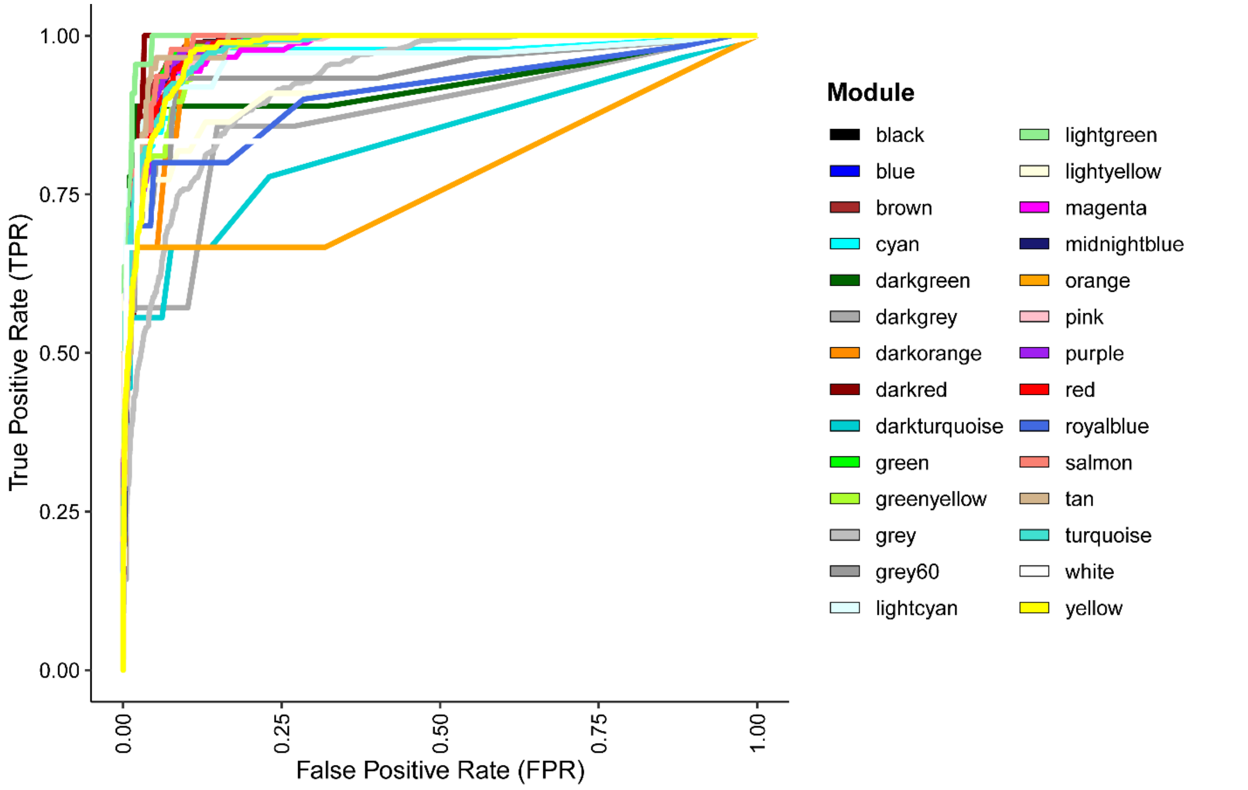

Figure S19. One-vs-rest ROC curves for each of the 28 modules in the test set. Each curve represents the true positive rate (TPR) versus false positive rate (FPR) for a specific module. The global area under the curve (AUC) was 0.949.

Table S8. Performance metrics of the Random Forest classifier for module assignment. Classification metrics including sensitivity, specificity, positive predictive value, negative predictive value, prevalence, detection rate, detection prevalence, and balanced accuracy are reported for each co-expression module identified by WGCNA.

Module (Class)	Sensitivity	Specificity	Pos Pred Value	Neg Pred Value	Prevalence	Detection Rate	Detection Prevalence	Balanced Accuracy
Class: black	0.74336	0.97807	0.73043	0.97945	0.07403	0.05503	0.07534	0.86072
Class: blue	0.80872	0.97241	0.76025	0.97917	0.09761	0.07894	0.10383	0.89057
Class: brown	0.83571	0.97367	0.76221	0.98325	0.09171	0.07665	0.10056	0.90469
Class: cyan	0.56521	0.99667	0.72222	0.99337	0.01506	0.00851	0.01179	0.78094
Class: darkgreen	0.33333	0.99901	0.5	0.99803	0.00294	0.00098	0.00196	0.66617
Class: darkgrey	0	0.99967	0	0.99770	0.00229	0	0.00032	0.49983
Class: darkorange	0.1666	1	1	0.99836	0.00196	0.00032	0.00032	0.58333
Class: darkred	0	1	NaN	0.99705	0.00294	0	0	0.5
Class: darkturquoise	0.11111	1	1	0.99737	0.00294	0.00032	0.00032	0.55555
Class: green	0.79149	0.97693	0.74104	0.98251	0.07697	0.06092	0.08221	0.88421
Class: greenyellow	0.5	0.993322	0.591837	0.99034	0.01899	0.00949	0.01605	0.74666
Class: grey	0.72606	0.92678	0.58209	0.96014	0.12316	0.08942	0.15362	0.82642
Class: grey60	0.3	1	1	0.99310	0.00982	0.00294	0.00294	0.65
Class: lightcyan	0.45945	0.999005	0.85	0.99340	0.01211	0.00556	0.00655	0.72923
Class: lightgreen	0.27272	0.99934	0.75	0.99474	0.00720	0.00196	0.00262	0.63603
Class: lightyellow	0.45454	0.99967	0.909091	0.99605	0.00720	0.00327	0.00360	0.72710
Class: magenta	0.59551	0.98718	0.58242	0.98785	0.02915	0.01736	0.02981	0.79134
Class: midnightblue	0.39130	0.996009	0.6	0.99073	0.015067	0.005896	0.009826	0.69365
Class: orange	0.16666	1	1	0.99836	0.001965	0.000327	0.000327	0.58333
Class: pink	0.77143	0.97749	0.71681	0.98302	0.06878	0.05306	0.07403	0.87446
Class: purple	0.52703	0.99362	0.67241	0.98831	0.02424	0.01277	0.019	0.76032
Class: red	0.75983	0.97557	0.71605	0.98043	0.07501	0.05699	0.07959	0.8677
Class: royalblue	0.3	0.9996714	0.75	0.9977042	0.0032755	0.0009826	0.0013102	0.6498357
Class: salmon	0.382979	0.997339	0.692308	0.99042	0.015395	0.005896	0.008516	0.690159

Class: tan	0.362069	0.995993	0.636364	0.987748	0.018998	0.006878	0.010809	0.679031
Class: turquoise	0.8048	0.97169	0.77681	0.976	0.10907	0.08778	0.113	0.88825
Class: white	0.1666667	1	1	0.9983617	0.0019653	0.0003275	0.0003275	0.5833333
Class: yellow	0.73818	0.97264	0.7276	0.97404	0.09008	0.06649	0.09139	0.85541

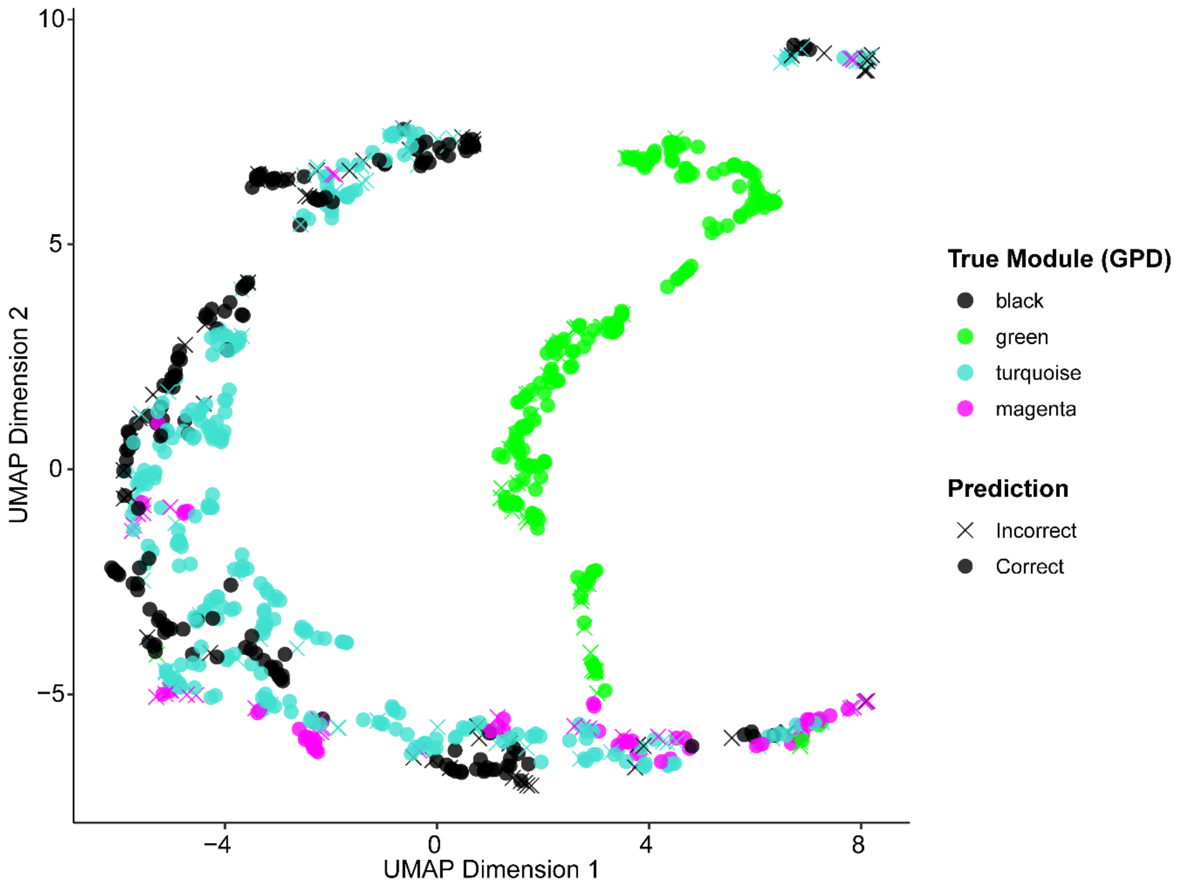

Figure S20. Two-dimensional UMAP projection of test genes belonging to the four *GPD*-associated modules (black, green, turquoise, and magenta). Points are colored by true module identity, and classification correctness is indicated by shape (cross = misclassified; circle = correct). UMAP was performed using n_neighbors = 5 and default settings for other parameters.

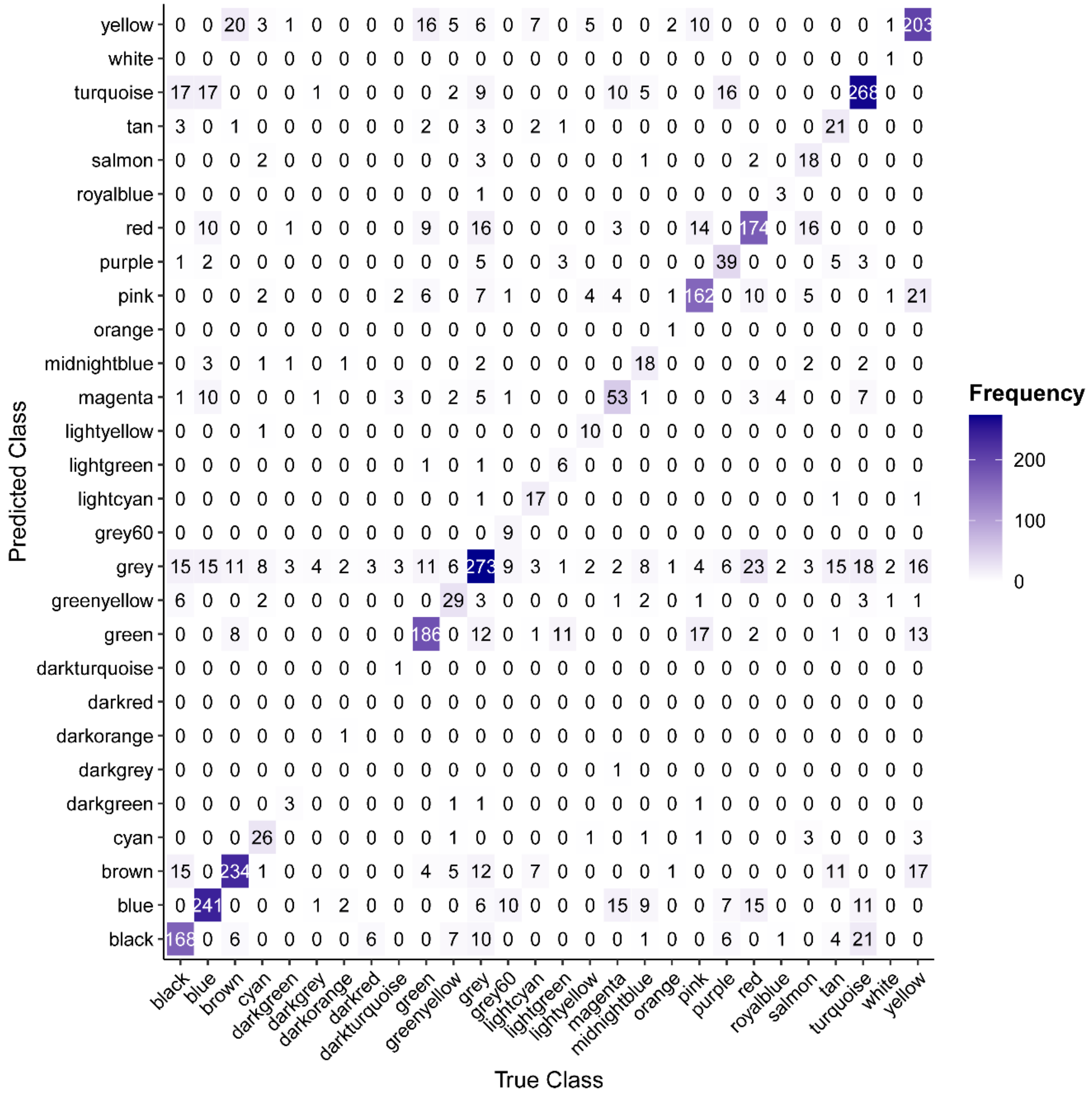

Figure S21.Confusion matrix showing classification performance of the Random Forest model across all 28 co-expression modules. The matrix compares true module labels (x-axis) with predicted labels (y-axis) for 3,053 test genes. The diagonal dominance reflects high agreement between predictions and true classes, particularly in modules with higher prevalence (e.g., turquoise, green, grey).
